# Supplementary material for: Rheological modification of partially oxidised cellulose nanofibril gels with inorganic clays
Source: PLoS One. 2021 Jul 7;16(7):e0252660. doi: 10.1371/journal.pone.0252660 (PMC8263268; doi:10.1371/journal.pone.0252660)
Supplement: S1 File — Frequency and amplitude sweep graphs, flow sweep curves, SAXS data and fitting parameters. (PDF) [file pone.0252660.s001.pdf]

Rheological modification of partially oxidised cellulose nanofibril gels with inorganic clays

Saffron J. Bryant,[1,2] Vincenzo Calabrese,[1] Marcelo A. da Silva,[1] Kazi M. Zakir Hossain,[1] Janet L. Scott,[1,3] Karen J. Edler[1*]*

[1] Department of Chemistry, University of Bath, Claverton Down, Bath, United Kingdom

[2] School of Science, RMIT University, Melbourne, Victoria, Australia

[3] Centre for Sustainable Chemical Technologies, University of Bath, Claverton Down, Bath, United Kingdom

* Corresponding authors:

Email: k.edler@bath.ac.uk, saffron.bryant@rmit.edu.au

S1. Supporting Information

Oxidised Cellulose Nanofibrils

The oxidised cellulose nanofibrils (OCNF) were prepared using TEMP/NaOCl/NaBr oxidation of wood pulp followed by high-pressure homogenisation following the standard method of Saito et al.[1, 2] The sample used in these experiments was provided as an 8wt% solids paste in water as a research sample from a commercial partner and has been described in detail elsewhere.[3] The degree of oxidation was determined by conductometric titration.[4]

Clay Characterisation

Dilute solutions (0.5 wt%) of the clays in DI water were used to measure the ζ-potential utilising a Malvern Zeta-sizer Nano ZSP® (Malvern, UK) in folded capillary electrode cells. Samples were equilibrated at 25°C for 120 s prior to testing and the results taken from an

average of 3 measurements of 100 scans each. This gave a ζ-potential of -24.9 mV for Laponite, and -29.1 mV for montmorillonite. This is in-keeping with previous work which recorded a ζ-potential of -28 mV for montmorillonite[5] but different to the reported literature value for Laponite which is around -40 mV.[6, 7]

Rheology

Dilute solutions (0.5 wt%) of the clays in DI water were used to measure the ζ-potential utilising a Malvern Zeta-sizer Nano ZSP® (Malvern, UK) in folded capillary electrode cells. Samples were equilibrated at 25°C for 120 s prior to testing and the results taken from an average of 3 measurements of 100 scans each. This gave a ζ-potential of -24.9 mV for Laponite, and -29.1 mV for montmorillonite. This is in-keeping with previous work which recorded a ζ-potential of -28 mV for montmorillonite[5] but different to the reported literature value for Laponite which is around -40 mV.[6, 7]

Frequency Sweeps

Figures S1 to S8 show the frequency sweep curves for all mixtures. The tan(δ) discussed in the paper was calculated by dividing G'' by G' at an angular frequency of 6.3 rad/s.

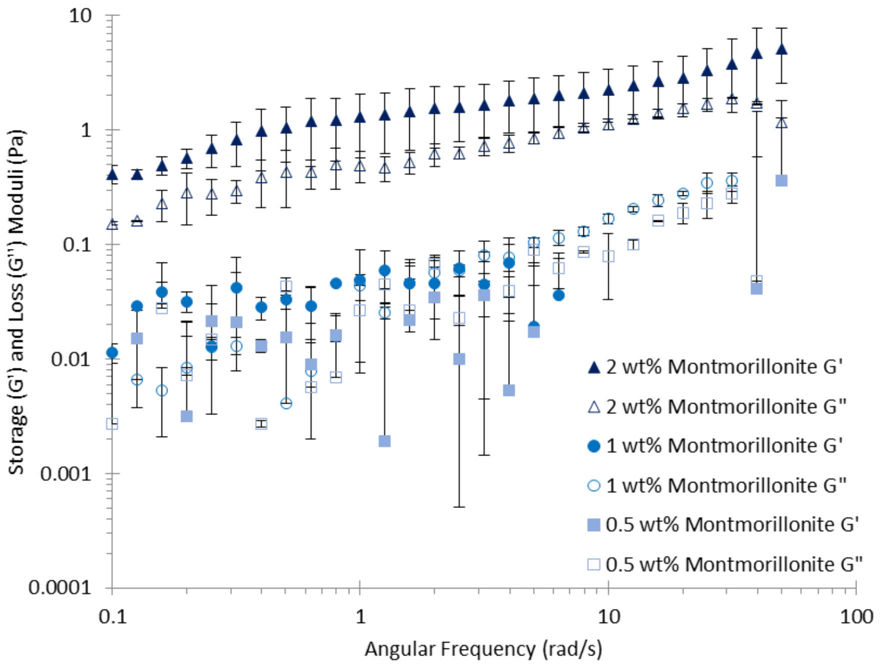

Figure S1. Frequency sweep of 0.5, 1, and 2 wt% montmorillonite in 0.75 wt% OCNF. This figure demonstrates the unreliability of measurements of these low-viscosity samples which were below the machine detection limits.

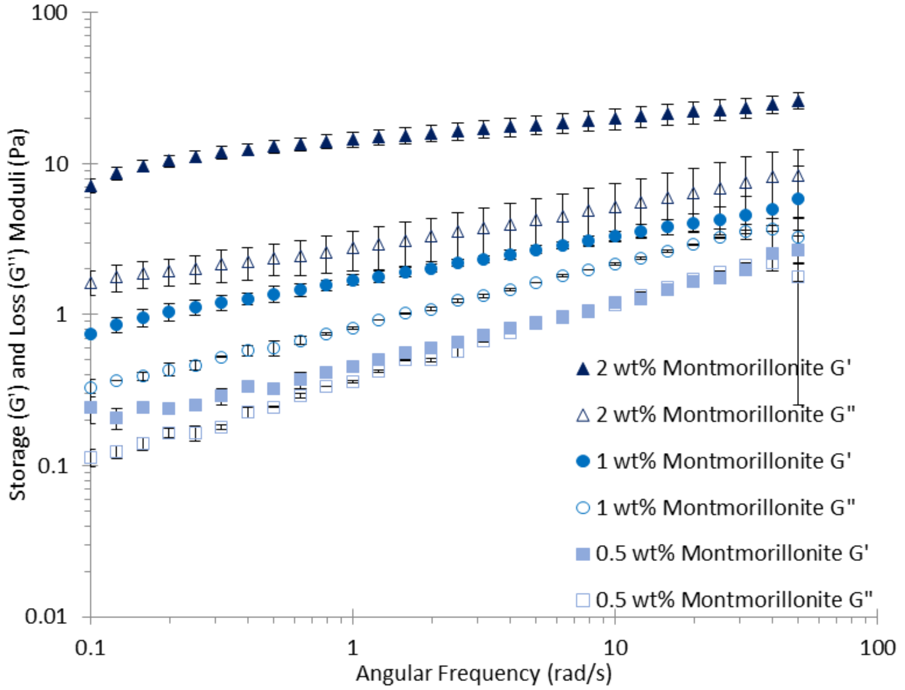

Figure S2. Frequency sweep of 0.5, 1, and 2 wt% montmorillonite in 1.5 wt% OCNF.

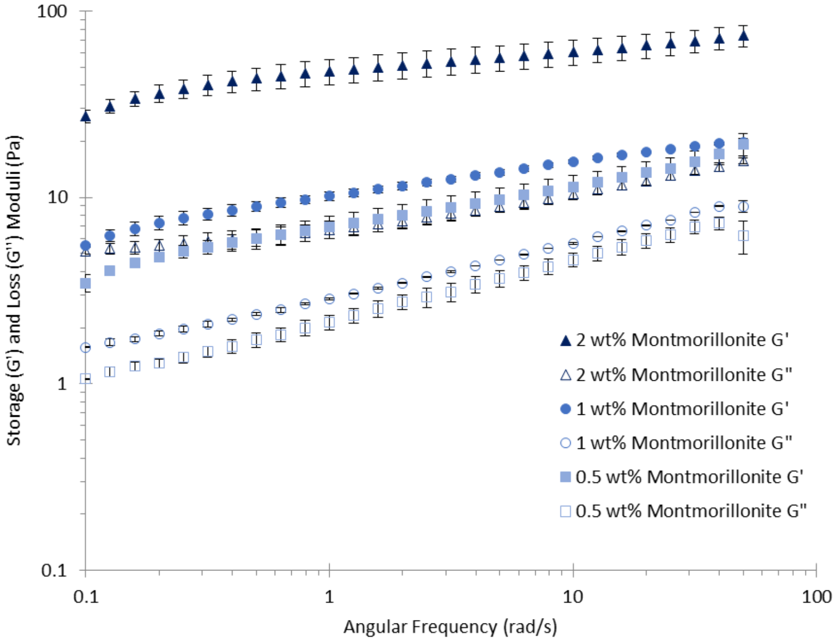

Figure S3. Frequency sweep of 0.5, 1, and 2 wt% montmorillonite in 2 wt% OCNF.

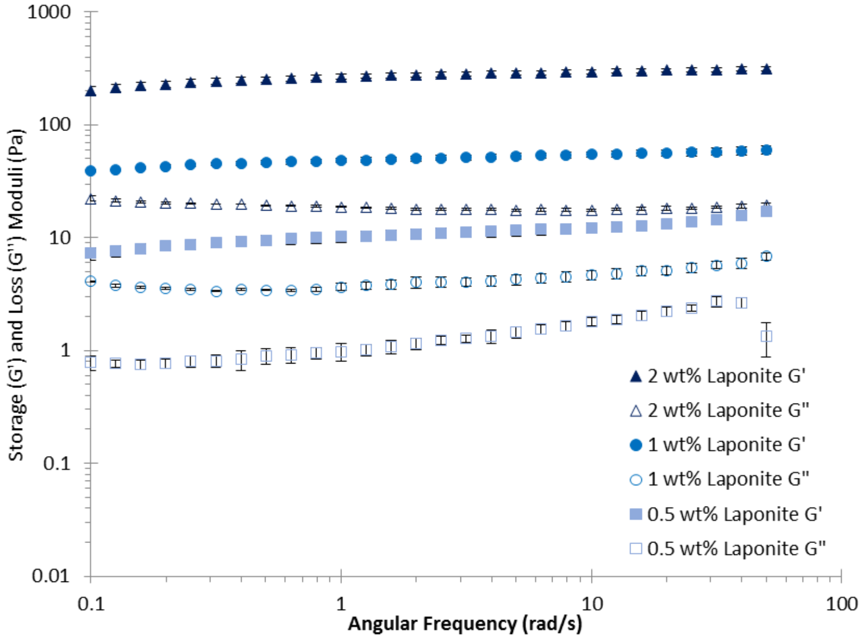

Figure S4. Frequency sweep of 0.5, 1, and 2 wt% laponite in 0.75 wt% OCNF.

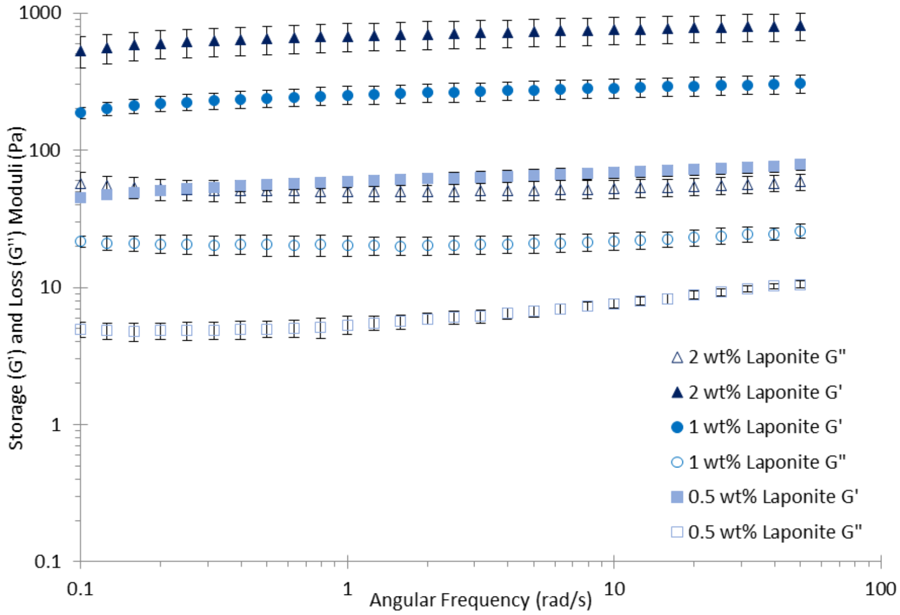

Figure S5. Frequency sweep of 0.5, 1, and 2 wt% laponite in 1.5 wt% OCNF.

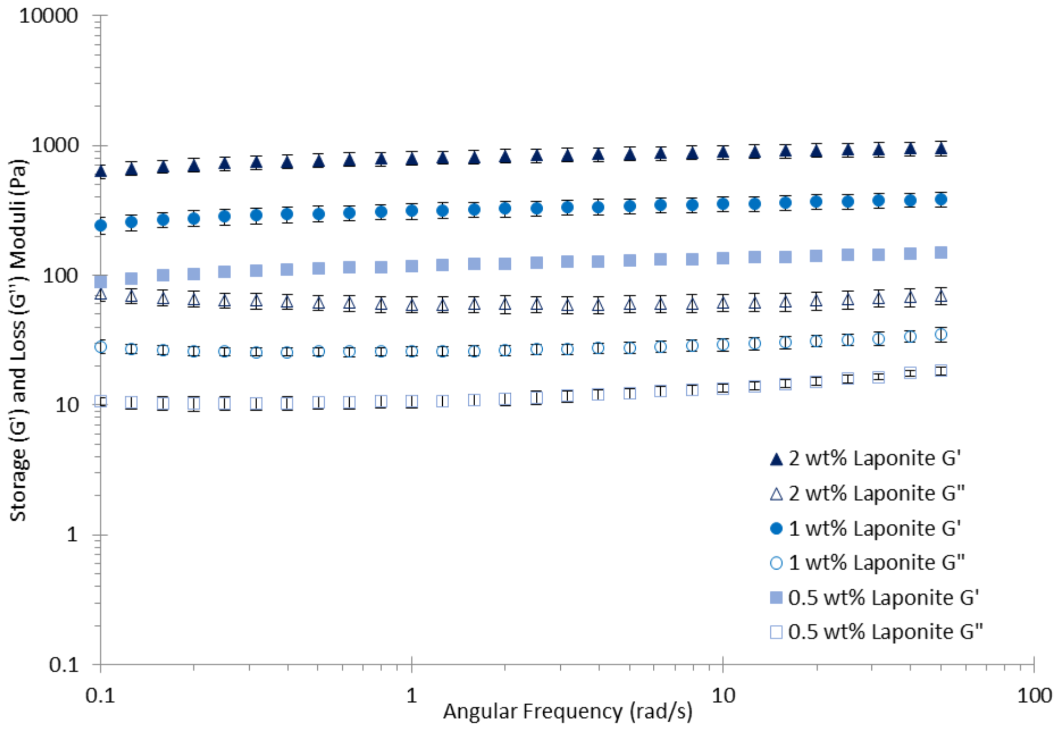

Figure S6. Frequency sweep of 0.5, 1, and 2 wt% laponite in 2 wt% OCNF.

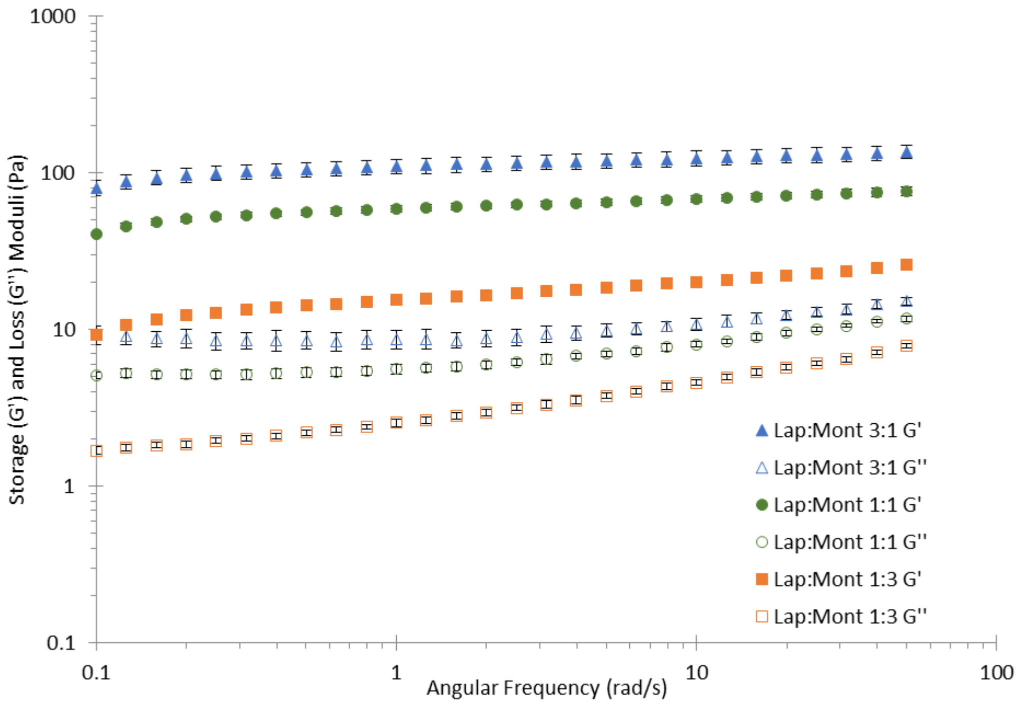

Figure S7. Frequency sweep of mixtures of laponite and montmorillonite in 1.5 wt% OCNF. Total clay concentration was 1 wt% for all samples.

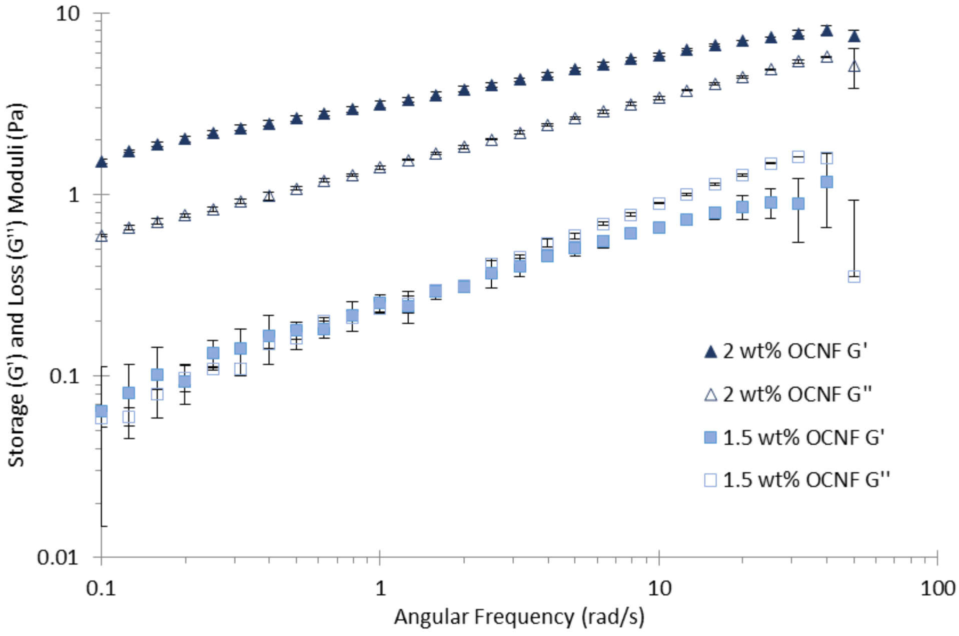

Figure S8. Frequency sweep of 1.5 wt% and 2 wt% OCNF.

Amplitude Sweep Curves

Figure S12 shows the amplitude sweep curves against both yield stress and yield strain as an example of the two data sets. The rest of the mixtures are only shown against yield stress but the same plots were made in order to calculate the yield stress values shown in the main manuscript.

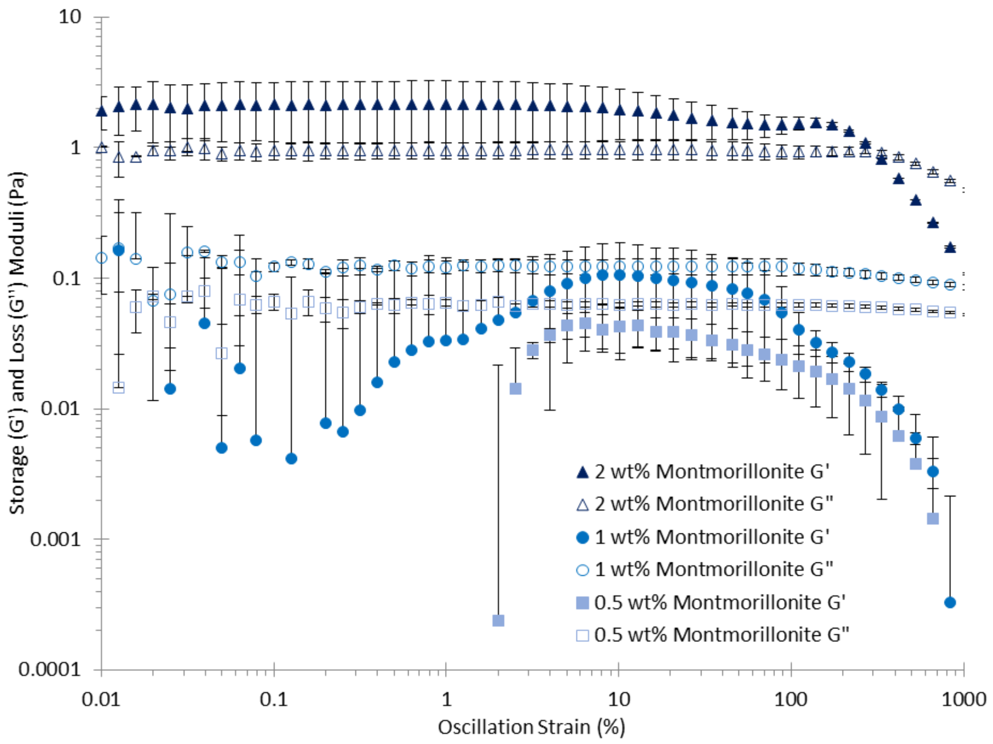

Figure S9. Amplitude sweep curves of 0.5, 1 and 2 wt% montmorillonite in 0.75 wt% OCNF. This figure demonstrates the noise and uncertainty of the data for samples with low concentrations of montmorillonite and OCNF.

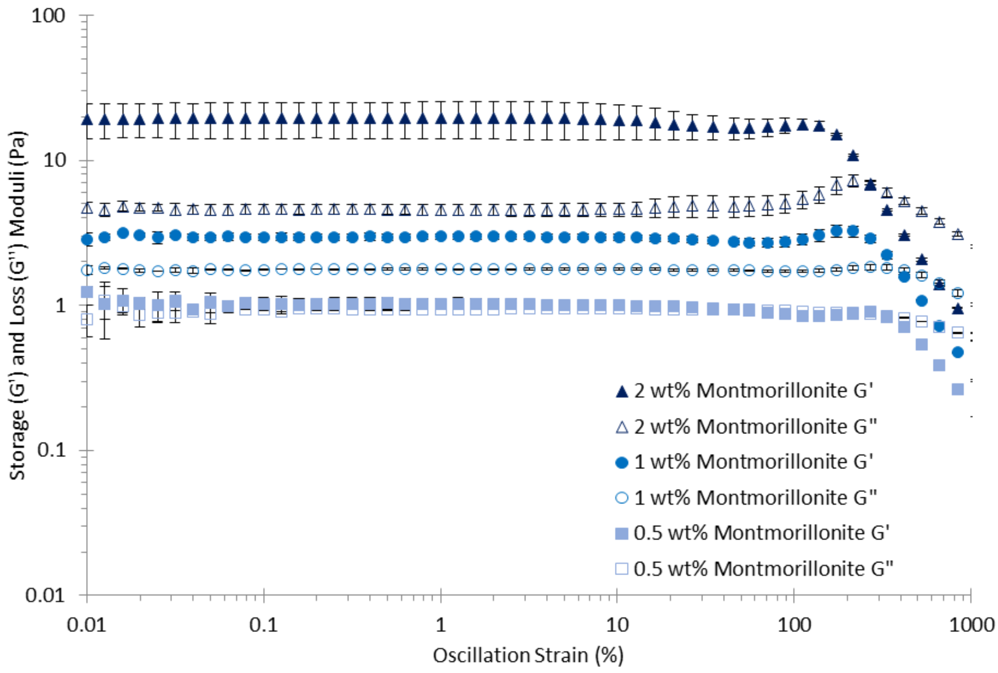

Figure S10. Amplitude sweep curves of 0.5, 1 and 2 wt% montmorillonite in 1.5 wt% OCNF.

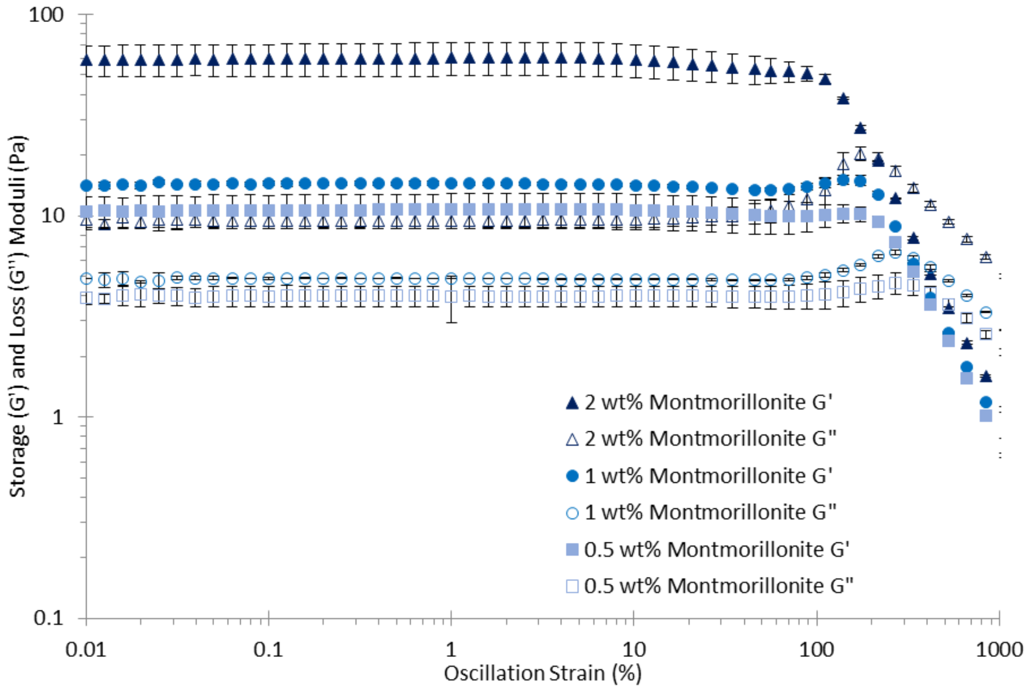

Figure S11. Amplitude sweep curves of 0.5, 1 and 2 wt% montmorillonite in 2 wt% OCNF

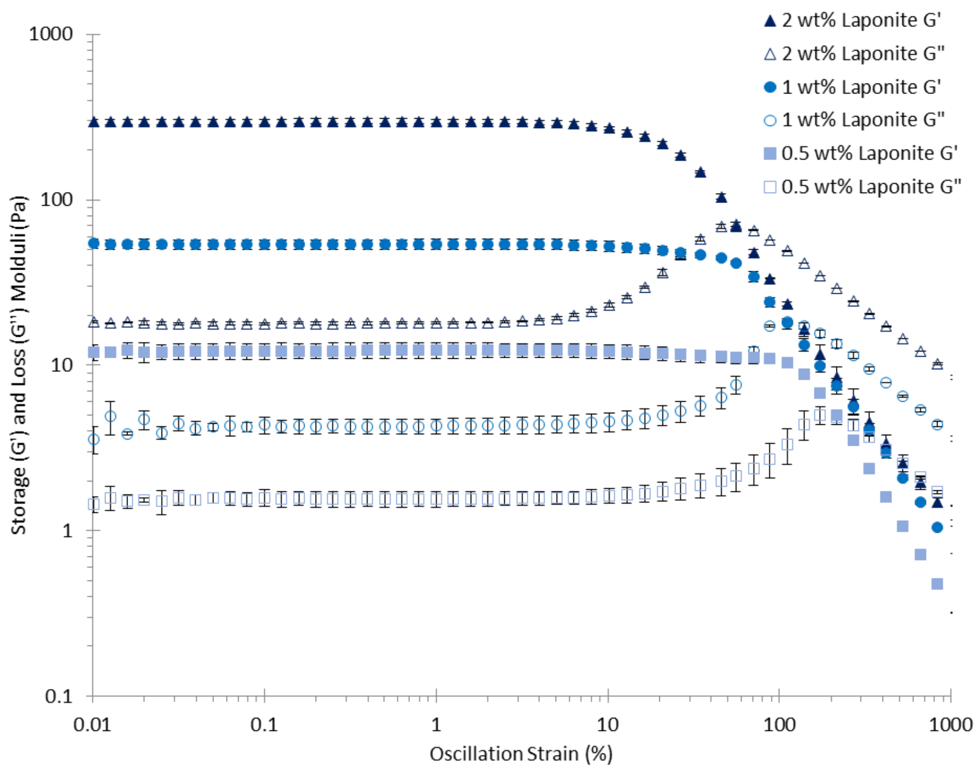

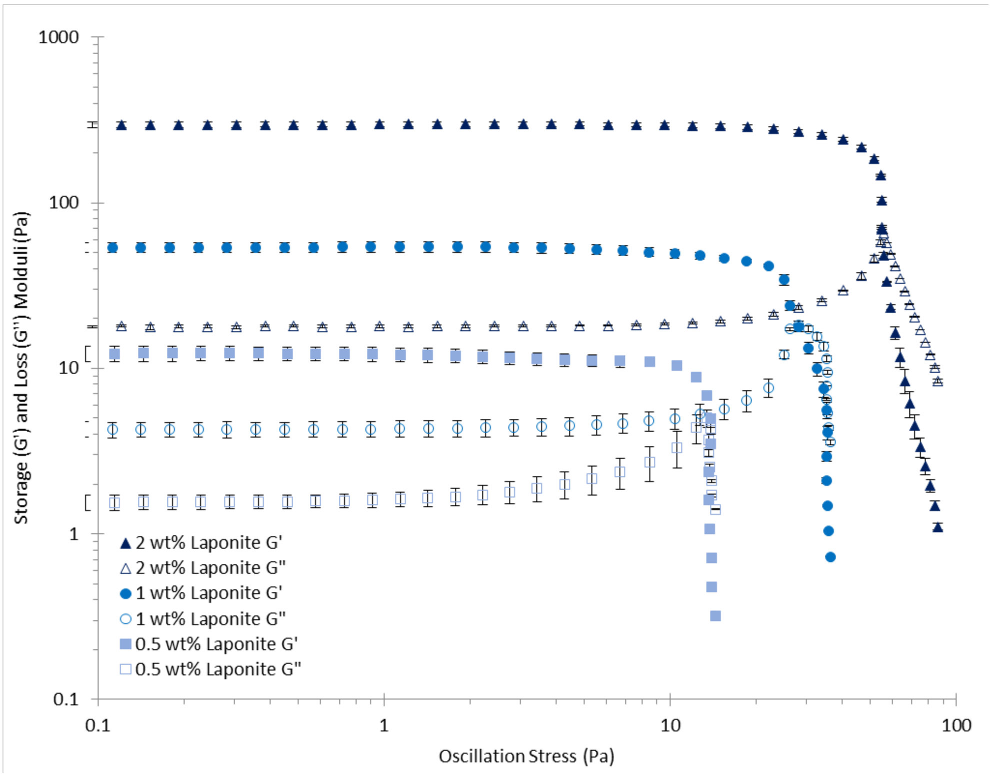

Figure S12. Amplitude sweep curves of 0.5, 1 and 2 wt% laponite in 0.75 wt% OCNF against strain (top) or stress (bottom).

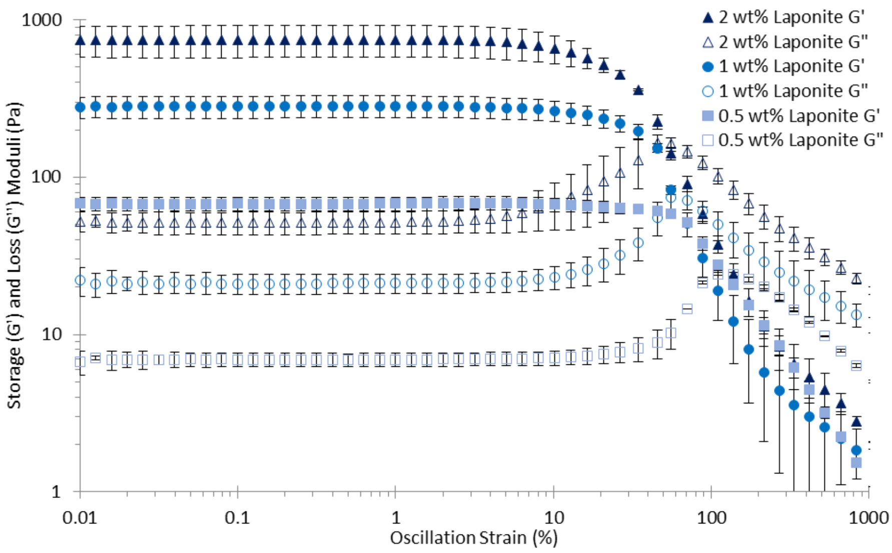

Figure S13. Amplitude sweep curves of 0.5, 1 and 2 wt% laponite in 1.5 wt% OCNF.

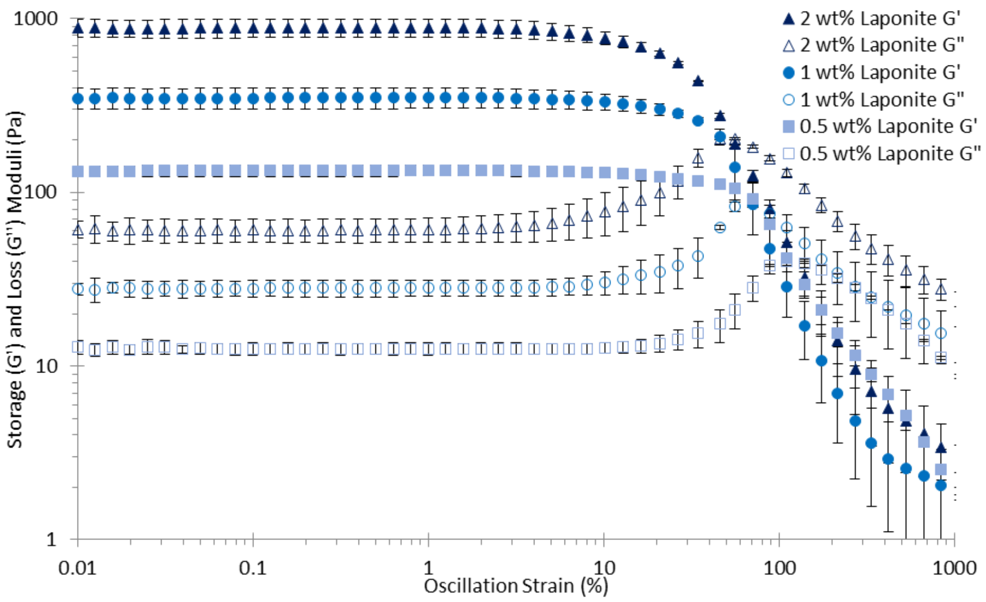

Figure S14. Amplitude sweep curves of 0.5, 1 and 2 wt% laponite in 2 wt% OCNF.

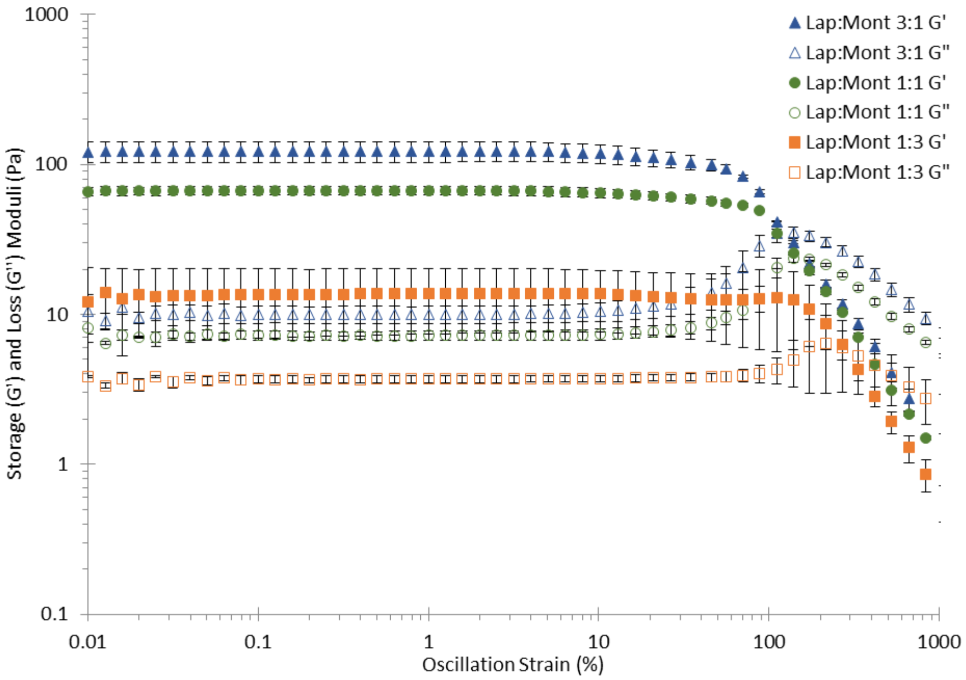

Figure S15. Amplitude sweep curves of laponite and montmorillonite mixtures in 1.5 wt% OCNF. All mixtures contain a total of 1 wt% clay.

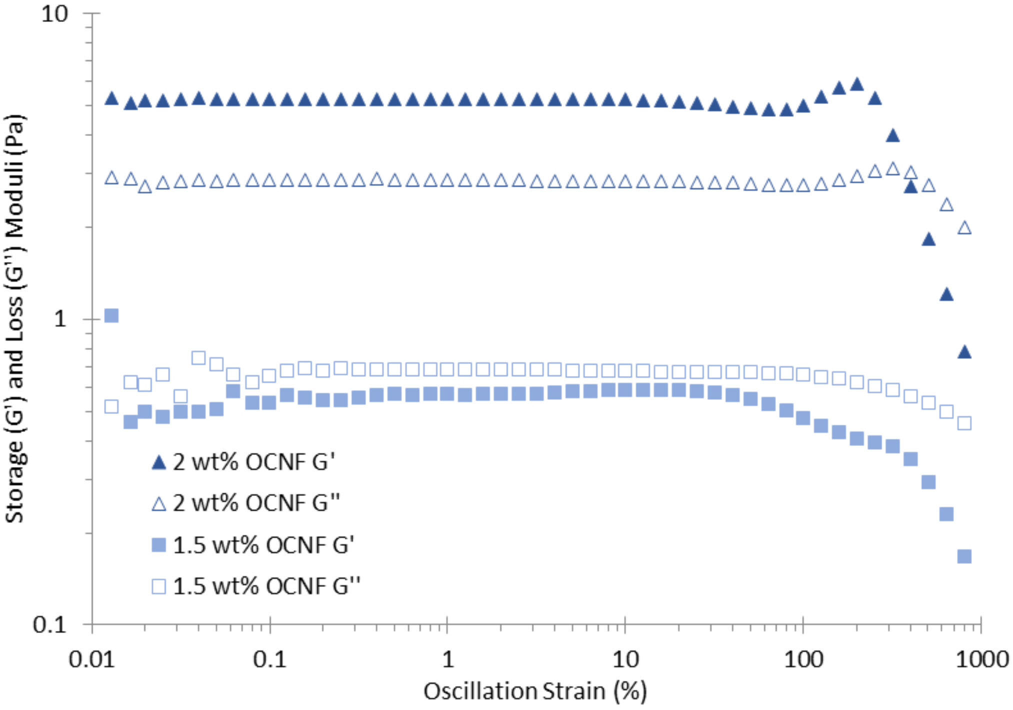

Figure S16. Amplitude sweep curves of 1.5 wt% and 2 wt % OCNF.

Flow Curves

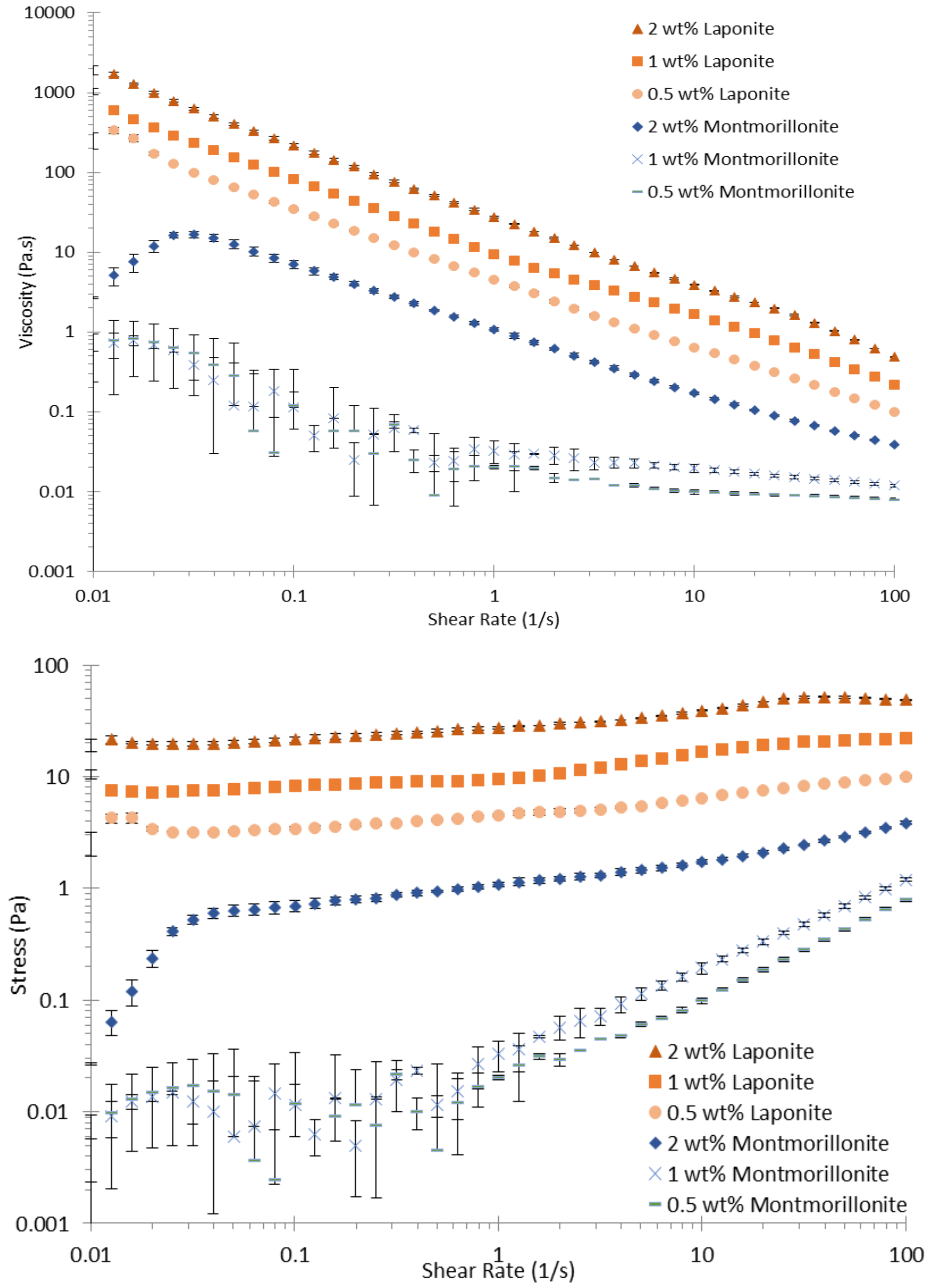

Figure S17. Flow sweep curves of laponite and montmorillonite in 0.75 wt% OCNF. Presented as shear rate against either viscosity (top) or stress (bottom).

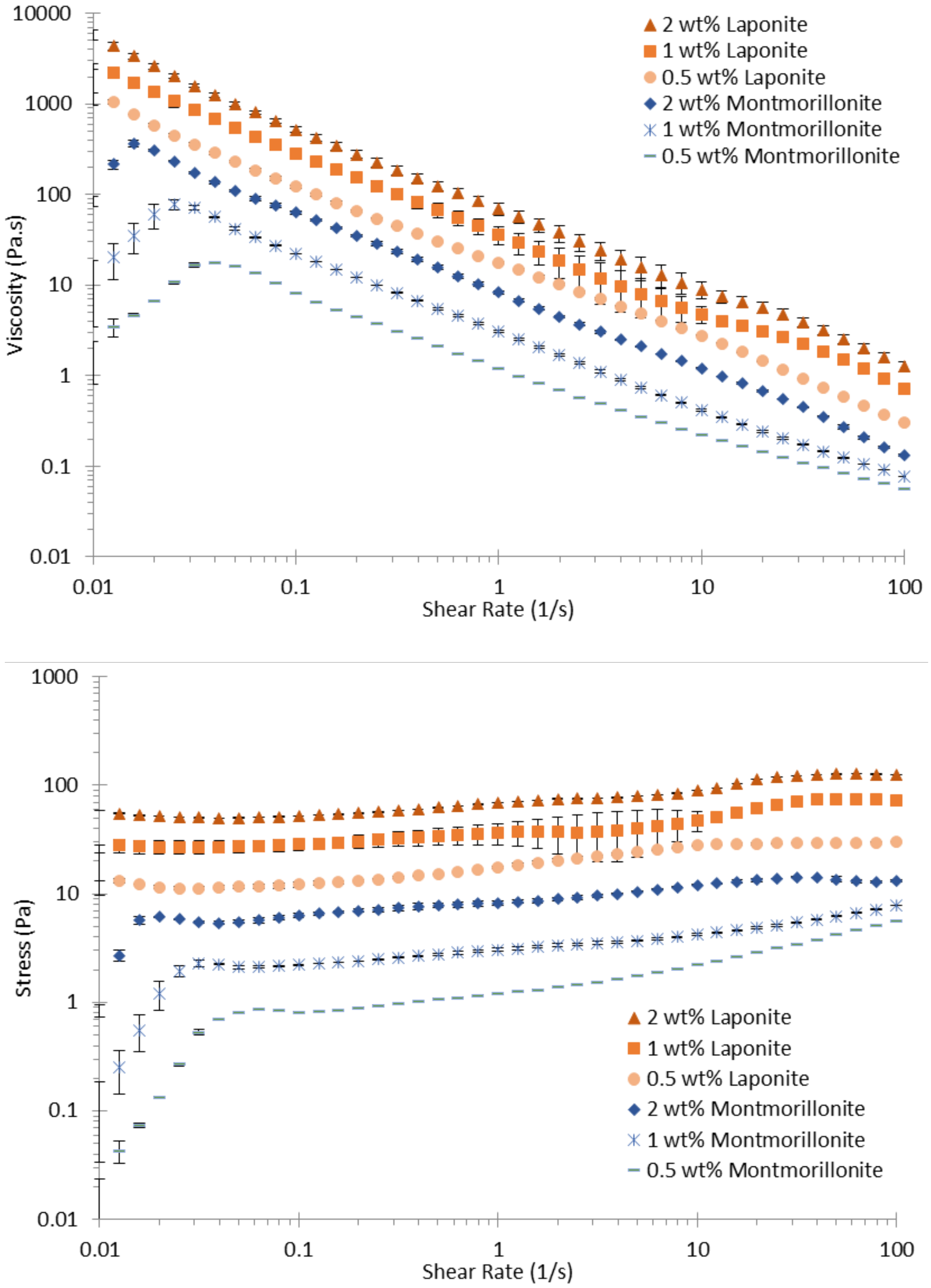

Figure S18. Flow sweep curves of laponite and montmorillonite in 1.5 wt% OCNF. Presented as shear rate against either viscosity (top) or stress (bottom).

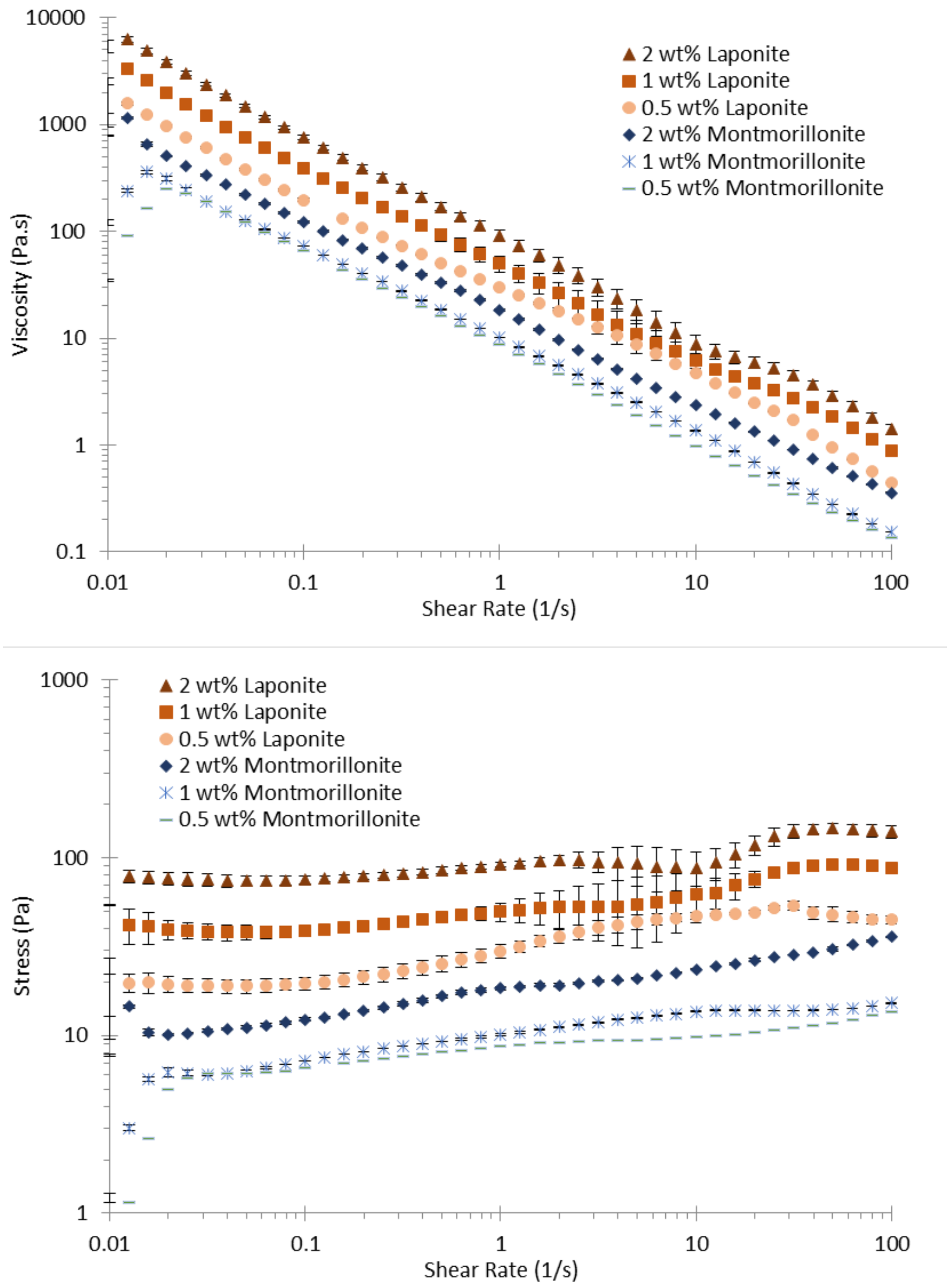

Figure S19. Flow sweep curves of laponite and montmorillonite in 2 wt% OCNF. Presented as shear rate against either viscosity (top) or stress (bottom).

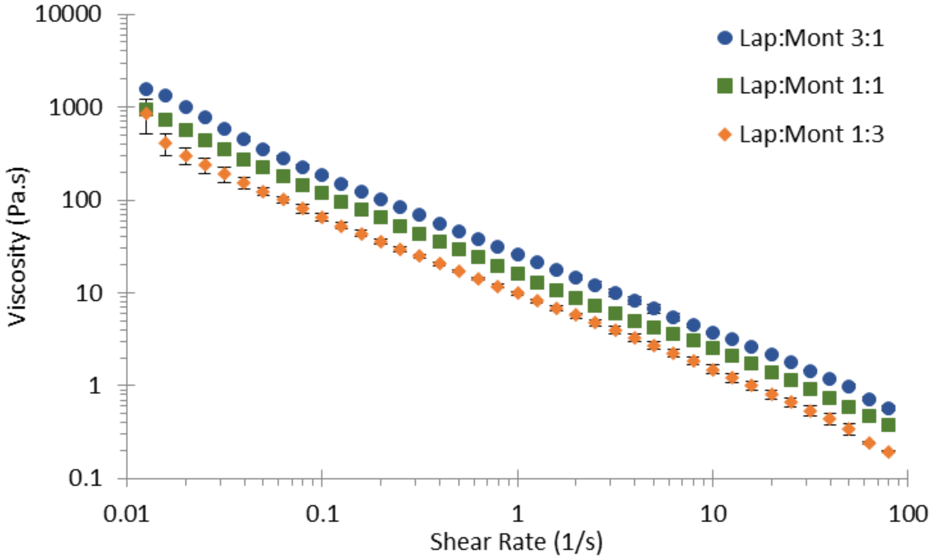

Figure S20. Flow sweep curve of mixtures of laponite and montmorillonite in 1.5 wt% OCNF. All mixtures contained a total of 1 wt% clay.

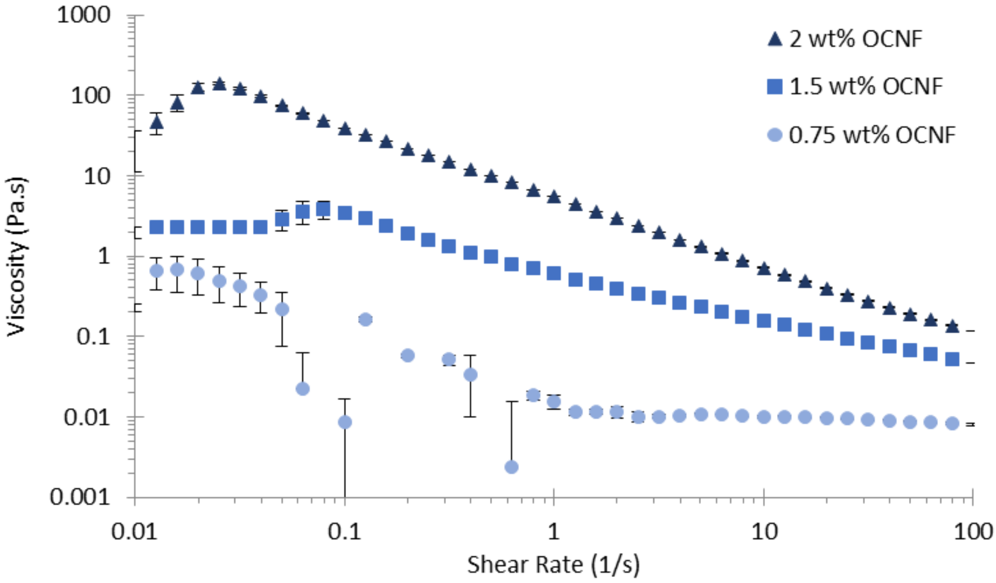

Figure S21. Flow sweep curve of 1.5 wt% and 2 wt% OCNF. Apparent missing data for 0.75 wt% OCNF were negative—a result of the equipment operating below its limits.

If a powerlaw is fit to the linear portion of the shear stress vs. shear rate curves (as shown in Figure S22) the resulting base and exponent provides quantitative information for comparing

the systems. Table S1 shows the power law fits for laponite and montmorillonite in different concentrations of OCNF, as well as for OCNF on its own.

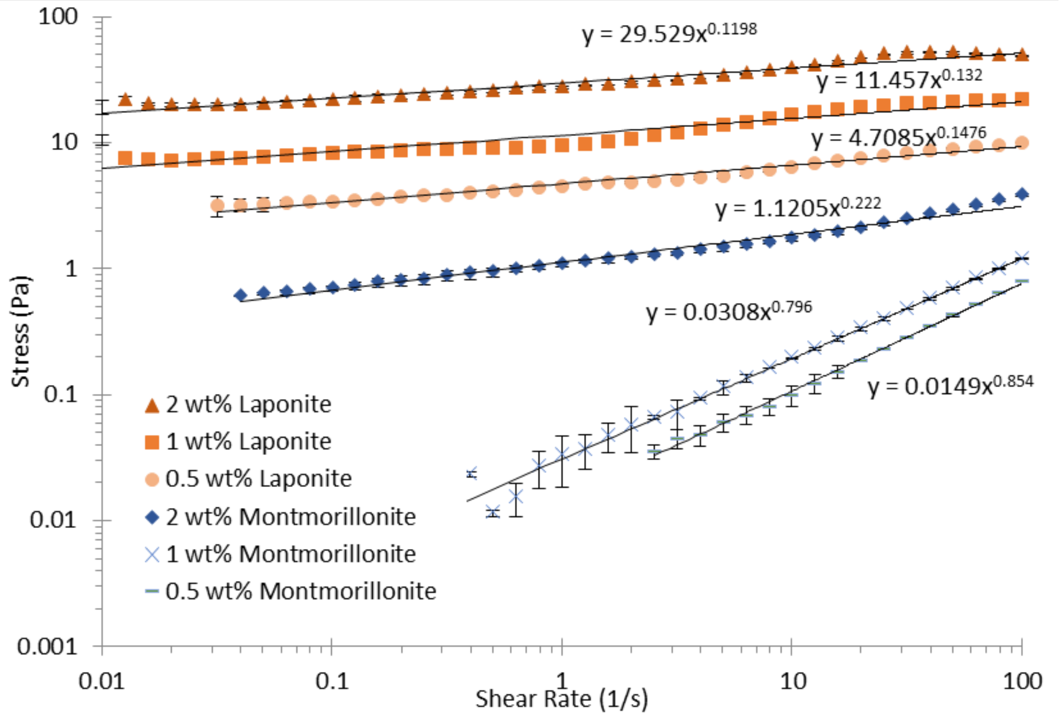

Figure S22. Linear portion of the flow sweep curves of laponite and montmorillonite in 1.5 wt% OCNF presented as shear rate against stress. Lines of best fit using a power law are shown in black, with the related equation.

The base number is the *flow consistency index* and provides information on the 'thickness' of the system, while the exponent is the *flow behaviour index* and provides information on the behaviour of the system under shear e.g. whether it is shear thinning and to what degree. A flow behaviour index of 1 means the system is Newtonian, >1 means the system is shear thickening, and <1 means the system is shear thinning.

Table S1. Power law fits for flow curves

	**Laponite**	**Montmorillonite**	**Laponite**	**Montmorillonite**
	Flow Consistency Index		Flow Behaviour Index	
**2 wt% OCNF**	5.4236		0.1434	
2 wt%	91.86	17.626	0.0767	0.1387
1 wt%	51.625	9.7093	0.1119	0.1168
0.5 wt%	29.932	8.278	0.1462	0.0918
**1.5 wt% OCNF**	0.5791		0.4398	
2 wt%	72.637	8.5269	0.1123	0.123
1 wt%	38.74	3.0853	0.1234	0.1644
0.5 wt%	18.128	1.2987	0.1347	0.2711
**0.75 wt% OCNF**	NA		NA	
2 wt%	29.529	1.1205	0.1198	0.222
1 wt%	11.457	0.0308	0.132	0.796
0.5 wt%	4.7085	0.0149	0.1476	0.854

Time-Sweeps

Figure S23 shows the oscillation curve of 1.5 wt% OCNF with either 1 wt% laponite or 1 wt% montmorillonite over the course of 72 hrs. For this measurement, strain was set to 0.1% and frequency was set to 6.28 rad/s. The measurement was performed at 25 °C with a thin layer of oil to prevent evaporation over the course of the experiment. The clay was added to OCNF immediately prior to placing into the rheometer, but as shown in the below figures, gel formation begins faster than the measurement can be started. However, there is a period of stabilization, then continuous gel-thickening over time. These results demonstrate that there is an aging effect in these gels, and for that reason all rheological measurements presented in this paper were carried out 24 hrs after combining the clays with OCNF. This is well after the initial exponential change in moduli, and allows consistency across samples.

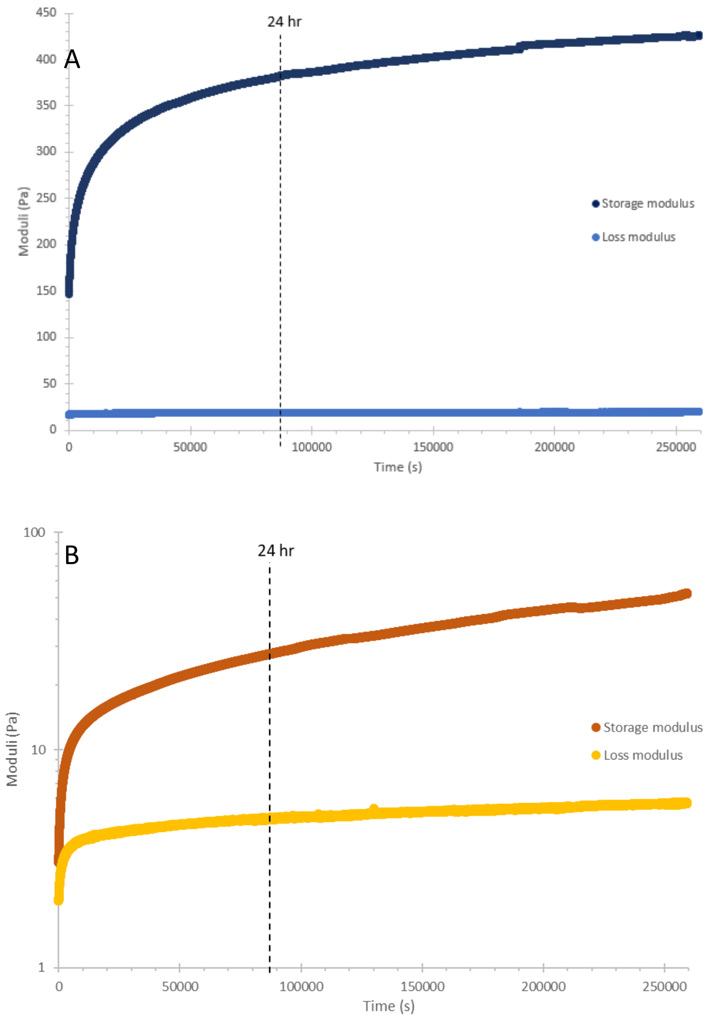

Figure S23. Time sweep of 1 wt% laponite (A), or 1 wt% montmorillonite (B) with 1.5 wt% OCNF.

SAXS

Figures S24 to S33 and Table S2 show SAXS data and corresponding best-fit parameters for clay and OCNF combinations. All fitting was done in SASView (Version 4.2.1, see http://www.sasview.org/ for more information). The models utilized were not modified from their implementation in SASView 4.2.1:

Cylinder – This fit is catalogued under “Cylinder Functions”.[8]

Elliptical Cylinder – This fit is catalogued under “Cylinder Functions”.[9]

Fitting was performed by fixing the SLDs of both the solvent and the particles at calculated values. The length of the OCNF cylinders was also fixed to 1000 Å as their true length is outside of the measurement range of this SAXS instrument. The background value for all fitting was fixed at the intensity value of the plateau at high q. For all fitting the scale factor was allowed to vary between 0.01 and 0.5 but in all cases ended being ~0.02-0.07.

For fitting the OCNF systems, the minimum radius and axis ratio were allowed to vary. For fitting the clay systems, both the length and the radius were allowed to vary. However, it was found that radii always fell above 250 Å for Laponite and 1000 Å for montmorillonite and did not affect the length. Therefore the maximum number of parameters fitted for any system was 3.

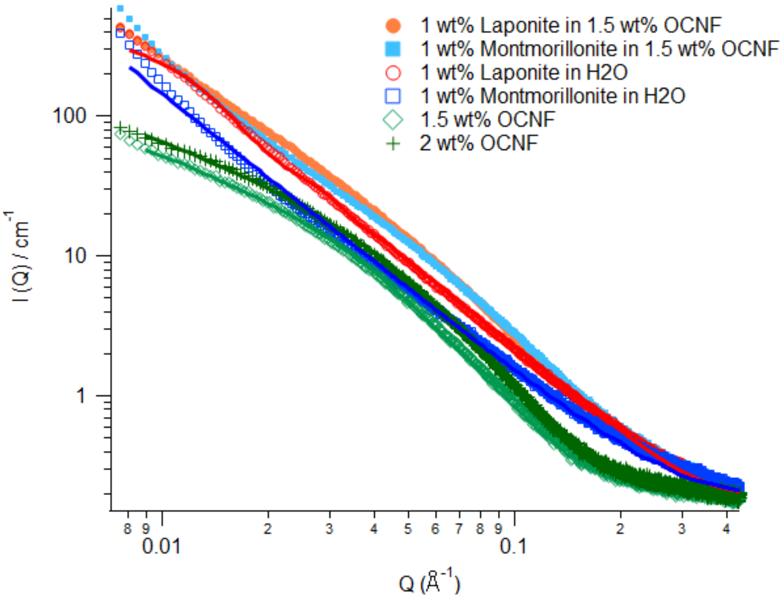

Figure S24. SAXS scattering patterns of 1 wt% laponite and montmorillonite in water and in 1.5 wt% OCNF, and 1.5 and 2 wt% OCNF on its own. Solid lines show fits (fitting parameters given in Table S2 below).

Table S2. Fitting parameters for models of best fit for 1.5 wt% OCNF and for laponite and montmorillonite in water. (Solvent SLD = 9.71 $\times 10^{-6}$)

**Sample**	**Model**	**Radius (Minor)**	**Axis Ratio**	**Length**	**SLD**
1.5 wt% OCNF	Elliptical cylinder	12.5±2	4.7±0.05	1000	13.6
2 wt% OCNF	Elliptical cylinder	12.3±2	4.4	1000	13.6
1 wt% Laponite in dH2O	Cylinder	≥250		11±2	22.3
1 wt% Montmorillonite + H2O	Cylinder	≥1000		10±2	19.6

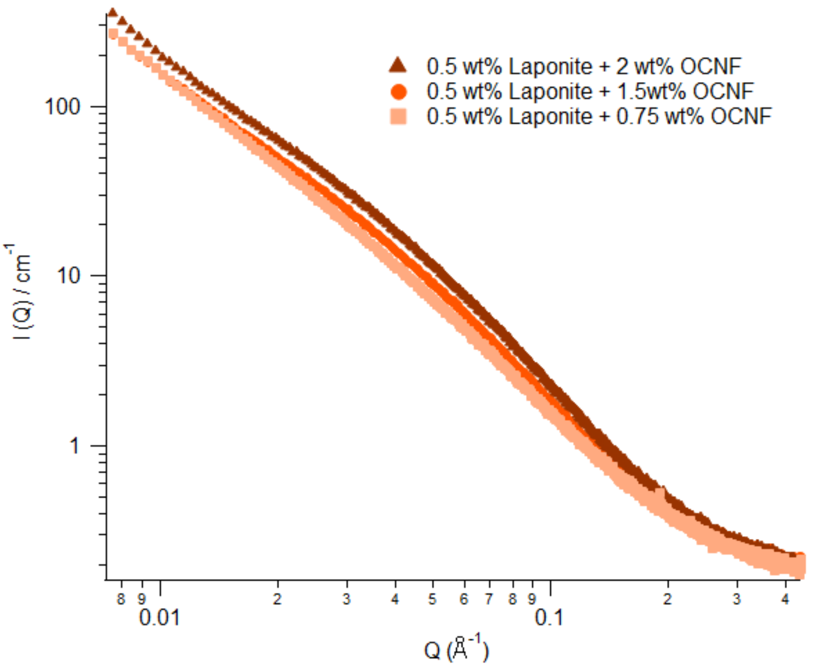

Figure S25. SAXS scattering patterns of 0.5 wt% laponite in 0.75, 1.5 and 2.0 wt% OCNF. Increasing the concentration of OCNF results in a more pronounced influence on the scattering pattern.

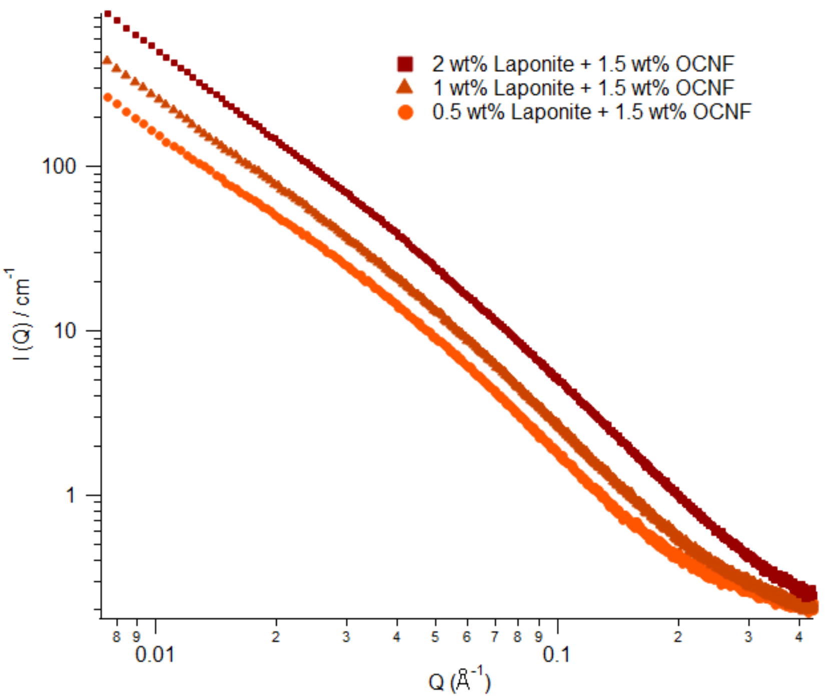

Figure S26. SAXS scattering patterns of 0.5, 1 and 2 wt% laponite in 1.5 wt% OCNF. Increasing the laponite concentration results in an increased intensity across the q range due to the increased volume of particles.

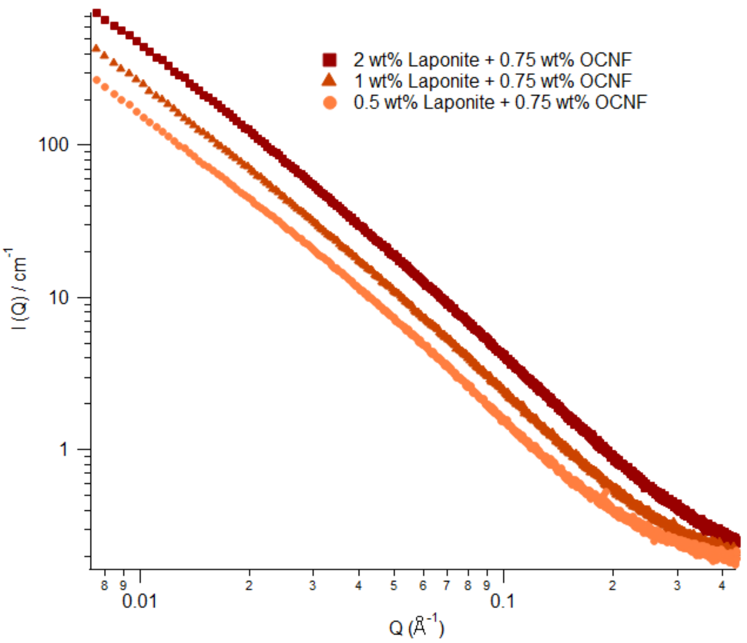

Figure S27. SAXS scattering patterns of 0.5, 1 and 2 wt% laponite in 0.75 wt% OCNF. Increasing the laponite concentration results in an increased intensity across the q range due to the increased volume of particles.

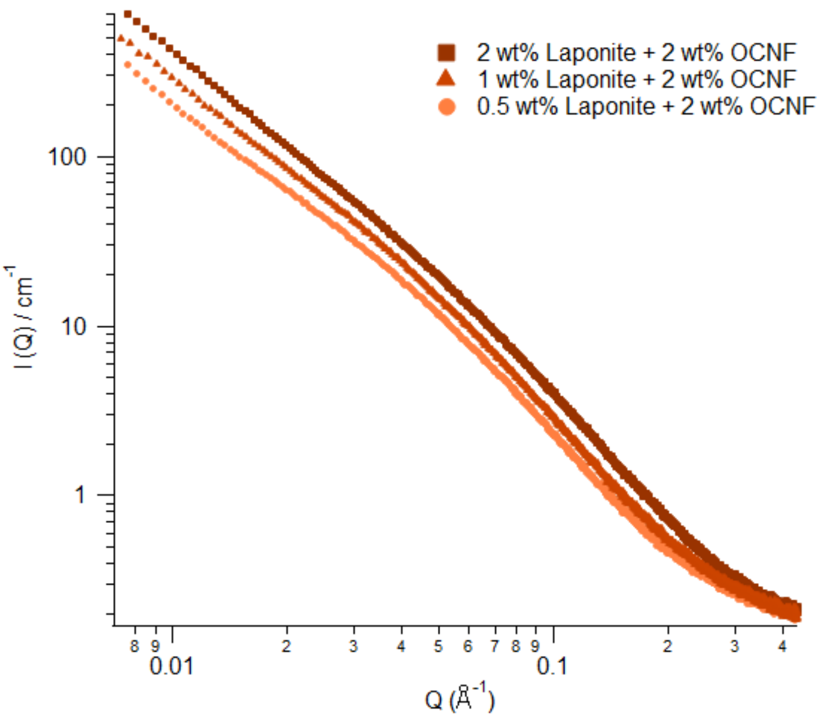

Figure S28. SAXS scattering patterns of 0.5, 1 and 2 wt% laponite in 2 wt% OCNF. Increasing the laponite concentration results in an increased intensity across the q range due to the increased volume of particles.

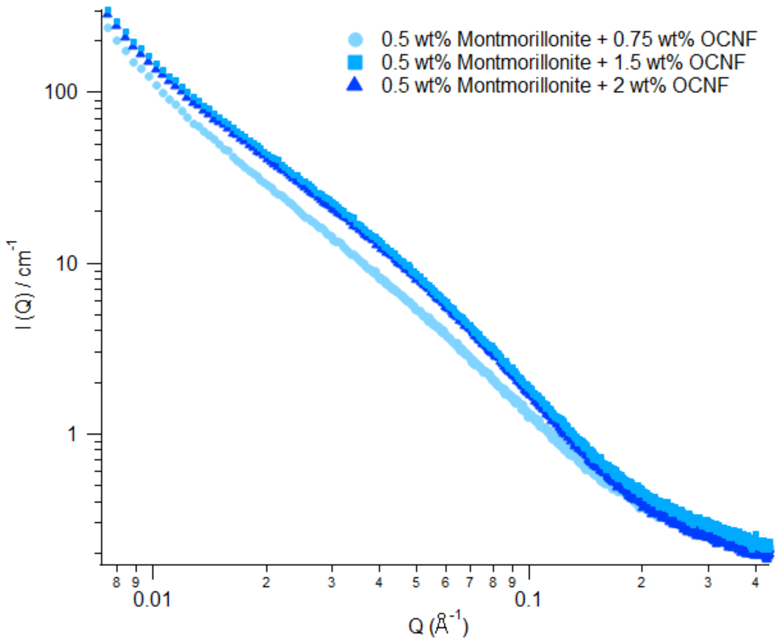

Figure S29. Scattering pattern of 0.5 wt% Montmorillonite in 0.75, 1.5 and 2 wt% OCNF. An increase from 0.75 wt% to 1.5 wt% OCNF results in a more pronounced influence of the OCNF pattern on the scattering curve. Between 1.5 and 2 wt% OCNF there is no change.

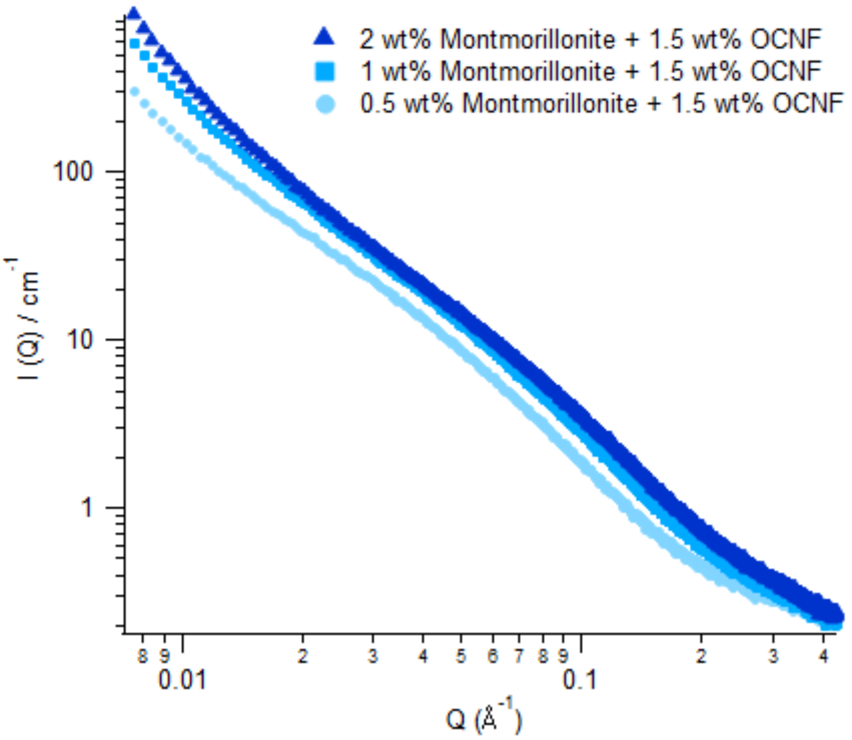

Figure S30. Scattering pattern of 0.5, 1, and 2 wt% Montmorillonite in 1.5 wt% OCNF. Increasing the montmorillonite concentration results in an increase in intensity across the Q range, probably due to an increase in the number of particles.

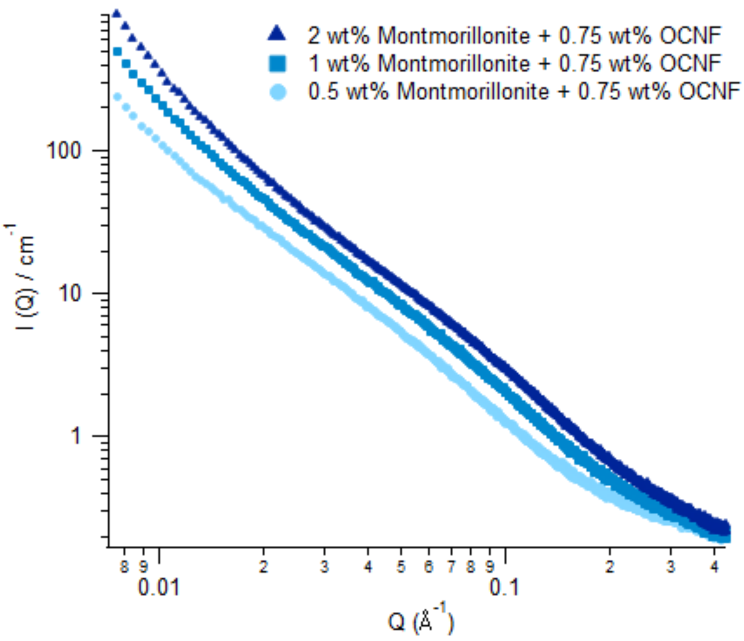

Figure S31. Scattering pattern of 0.5, 1, and 2 wt% Montmorillonite in 0.75 wt% OCNF. Increasing the montmorillonite concentration results in an increase in intensity across the Q range, probably due to an increase in the number of particles.

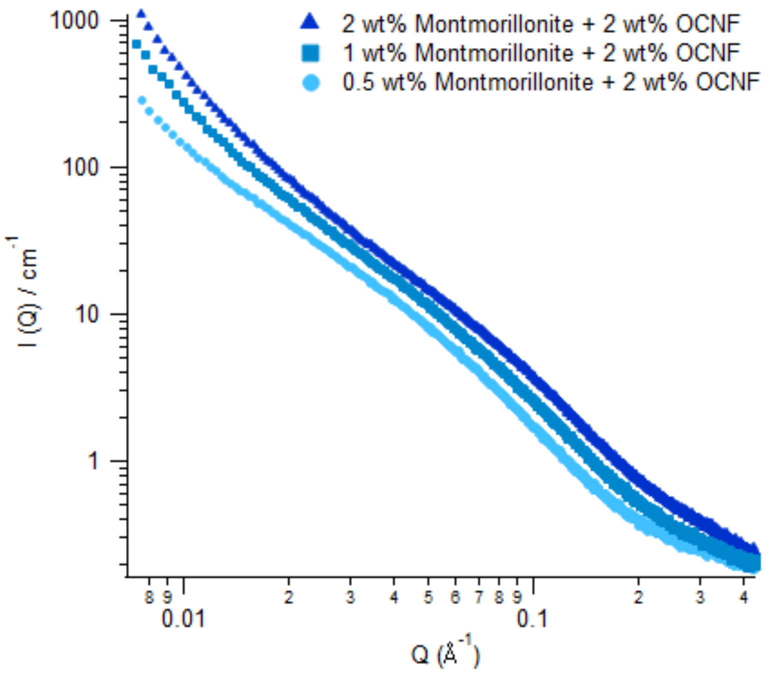

Figure S32. Scattering pattern of 0.5, 1, and 2 wt% Montmorillonite in 2 wt% OCNF. Increasing the montmorillonite concentration results in an increase in intensity across the Q range, probably due to an increase in the number of particles.

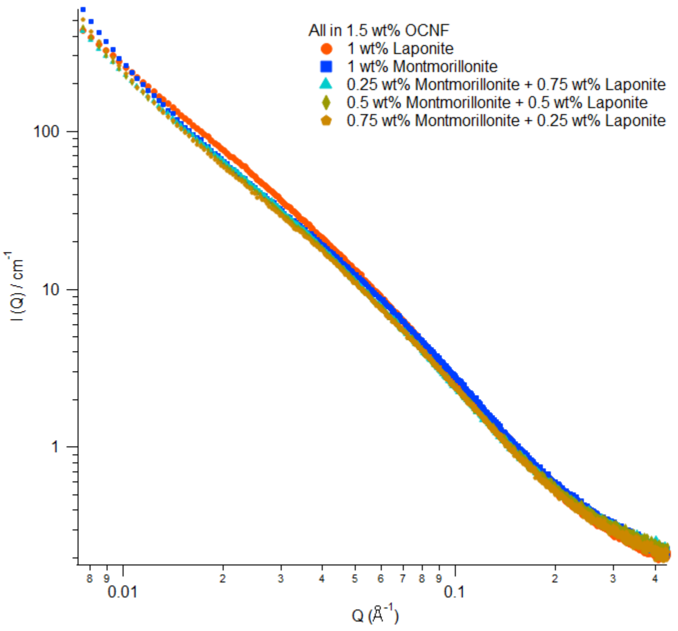

Figure S33. SAXS patterns of laponite and montmorillonite at different ratios in 1.5 wt% OCNF. There was little to no difference in the scattering curves between pure laponite or montmorillonite and mixtures of the two. This is expected because the only real difference is that the montmorillonite particles have a much bigger radius which is outside this q-range.

Visual Observation

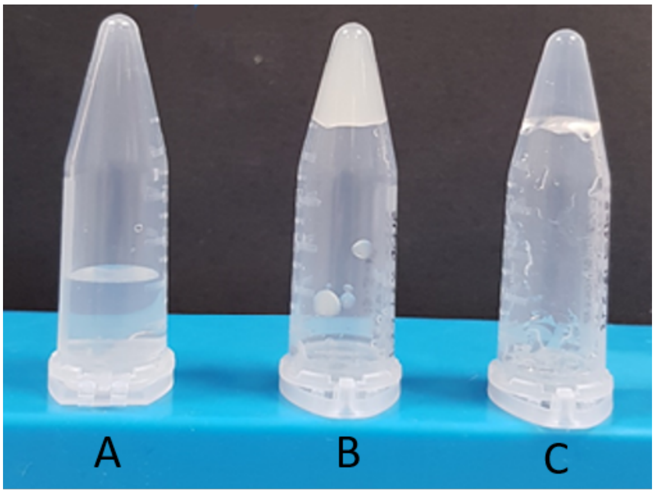

Figure S34. Shows the flow properties of 1.5 wt% OCNF on its own (A), and with 2 wt% montmorillonite (B) or 2 wt% laponite (C).

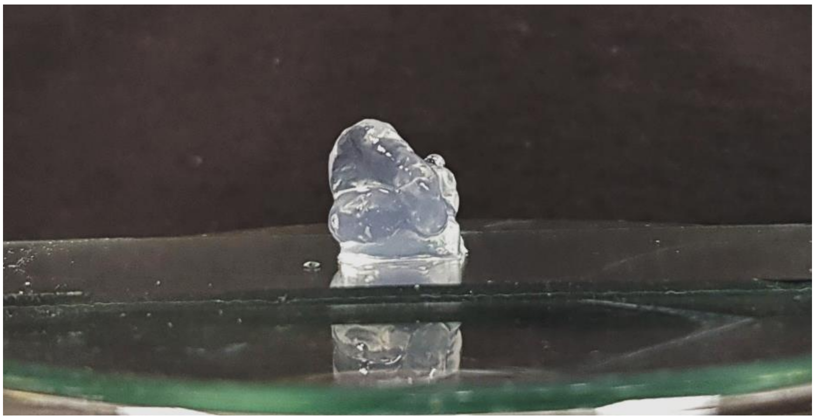

Figure S35. Shows the self-standing nature of a gel made from 1.5 wt% OCNF and 2 wt% Laponite.

References

1. Saito T, Nishiyama Y, Putaux J-L, Vignon M, Isogai A. Homogeneous Suspensions of Individualized Microfibrils from TEMPO-Catalyzed Oxidation of Native Cellulose. Biomacromolecules. 2006;7(6):1687-91. doi: 10.1021/bm060154s.

2. Isogai A, Saito T, Fukuzumi H. TEMPO-oxidized cellulose nanofibers. Nanoscale. 2011;3(1):71-85. doi: 10.1039/C0NR00583E.

3. Schmitt J, Calabrese V, da Silva MA, Lindhoud S, Alfredsson V, Scott JL, et al. TEMPO-oxidised cellulose nanofibrils; probing the mechanisms of gelation via small angle X-ray scattering. Physical Chemistry Chemical Physics. 2018;20(23):16012-20. doi: 10.1039/C8CP00355F.

4. Courtenay JC, Johns MA, Galembeck F, Deneke C, Lanzoni EM, Costa CA, et al. Surface modified cellulose scaffolds for tissue engineering. Cellulose. 2017;24(1):253-67. doi: 10.1007/s10570-016-1111-y.

5. Liu Y, Gordeyeva K, Bergström L. Steady-shear and viscoelastic properties of cellulose nanofibril–nanoclay dispersions. Cellulose. 2017;24(4):1815-24. doi: 10.1007/s10570-017-1211-3.

6. Xiong Z-Q, Li X-D, Fu F, Li Y-N. Performance evaluation of laponite as a mud-making material for drilling fluids. Petroleum Science. 2019;16(4):890-900. doi: 10.1007/s12182-018-0298-y.

7. Brunier B, Sheibat-Othman N, Chniguir M, Chevalier Y, Bourgeat-Lami E. Investigation of Four Different Laponite Clays as Stabilizers in Pickering Emulsion Polymerization. Langmuir. 2016;32(24):6046-57. doi: 10.1021/acs.langmuir.6b01080.

8. Pedersen JS. Analysis of small-angle scattering data from colloids and polymer solutions: modeling and least-squares fitting. Advances in Colloid and Interface Science. 1997;70:171-210. doi: https://doi.org/10.1016/S0001-8686(97)00312-6.

9. Feigin LA, Svergun DI. Structure Analysis by Small-Angle X-Ray and Neutron Scattering: Springer US; 1987. 335 p.
