# Supplementary material for: Rheological modification of partially oxidised cellulose nanofibril gels with inorganic clays
Source: PLoS One. 2021 Jul 7;16(7):e0252660. doi: 10.1371/journal.pone.0252660 (PMC8263268; doi:10.1371/journal.pone.0252660)
Supplement: Attachment — Submitted filename: response to reviewers - PONE-D-20-31757R1.pdf [file pone.0252660.s002.pdf]

UNIVERSITY OF BATH

Prof. Karen J. Edler
Department of Chemistry,
Bath BA2 7AY • United Kingdom

Telephone 01225 384192
Email: K.Edler@bath.ac.uk

13 January 2021

Dear Pratheep K. Annamalai,

Thank you for giving us the opportunity to resubmit our manuscript titled “Rheological modification of partially oxidised cellulose nanofibril gels with inorganic clays” with feedback from the reviewers.

We appreciate their insightful comments and have addressed them below (our responses are shown in red). We have also taken your thoughtful feedback on board and made appropriate changes, especially in regards to meeting the formatting requirements of PLOS ONE.

We hope that the paper is now ready for publication in PLOS ONE and are confident that it will be of interest to your readership.

Yours sincerely,

Karen J Edler

Karen J Edler

Professor of Soft Matter

Reviewer and Editor Comments (Author responses in red)

This manuscript reports the rheological (gelation) behavior of oxidised cellulose nanofibre inluenced by two different types of clays which present platelet-like morphology. This is an important contribution to the field of nano-materials and applications, and suitable for the journal.

Before this can be considered for publication, it needs revision according to the reviewers' comments.

1. It would be good to add the detailed description in Supporting information on the preparation and characterisation of oxidised nanofibre (including source, experimental conditions, yield).

A section has been added to page 1 of the SI to describe the method of preparation and characterisation of the OCNF. This has now also been mentioned in the Methods section, line 97.

2. Were there any further characterizations performed on nanoclays such as CEC, pH in water or other that would be relevant for this study (apart from the platelet size). Ionic charges and strength on the surface might influence the interactions between nanofibers and clays. Correlations with such details in the discussion will enhance the clarity on structural information as indicated in reviewer comments.

The ζ-potential of each clay were measured dispersed in water and found to be similar. This information has been added to the SI and a sentence added to the main manuscript (line 244-45). Further characterisation of the materials was not performed as they are commercially available and characterised elsewhere.

3. Graphical representations of interactions (stable dispersion vs agglomeration) between nanofibres and two different clays (30 nm vs 300 nm) would be useful for the discussions.

Figure 7 has been added which shows interaction of the two differently sized particles with the OCNF fibrils.

4. Please ensure that your manuscript meets PLOS ONE's style requirements, including those for file naming. The PLOS ONE style templates can be found at

https://journals.plos.org/plosone/s/file?id=wjVg/PLOSOne_formatting_sample_main_body.pdf and

https://journals.plos.org/plosone/s/file?id=ba62/PLOSOne_formatting_sample_title_authors_affiliations.pdf

The manuscript has been modified to meet the style requirements including renaming the figures and changing the heading styles.

5. We note that you have provided funding information that is not currently declared in your Funding Statement. However, funding information should not appear in the Acknowledgments section or other areas of your manuscript. We will only publish funding information present in the Funding Statement section of the online submission form.

Please remove any funding-related text from the manuscript and let us know how you would like to update your Funding Statement. Currently, your Funding Statement reads as follows:

"KJE (principal investigator) and JLS (co-investigator) received an award from the Engineering and Physical Sciences Research Council to fund this project, which employed SB, MADS and KMZH (grant no. EP/N033310/1) https://epsrc.ukri.org/. The funders had no role in study design, data collection and analysis, decision to publish, or preparation of the manuscript."

Please include your amended statements within your cover letter; we will change the online submission form on your behalf.

Thank you for bringing this to our attention. The funding information has been removed from the acknowledgements section.

Can you please modify the Funding Statement to read:

KJE (principal investigator) and JLS (co-investigator) received an award from the Engineering and Physical Sciences Research Council to fund this project, which employed SB, MADS and KMZH (grant no. EP/N033310/1) https://epsrc.ukri.org/. VC received funding from the University of Bath to support his PhD candidature. The funders had no role in study design, data collection and analysis, decision to publish, or preparation of the manuscript.

6. We note that you have stated that you will provide repository information for your data at acceptance. Should your manuscript be accepted for publication, we will hold it until you provide the relevant accession numbers or DOIs necessary to access your data. If you wish to make changes to your Data Availability statement, please describe these changes in your cover letter and we will update your Data Availability statement to reflect the information you provide.

   Thank you. The data will be available here: https://doi.org/10.15125/BATH-00791 once the article is accepted for publication.

7. Please include captions for your Supporting Information files at the end of your manuscript, and update any in-text citations to match accordingly. Please see our Supporting Information guidelines for more information: http://journals.plos.org/plosone/s/supporting-information.

   Captions for all of the supporting information files have been added to the end of the manuscript. In addition, the SI files containing figures and tables have been saved separately.

Reviewer #1: The manuscript study the modification of the rheological properties of partially oxidised cellulose nanofibrils (OCNF) by the addition of Laponite or montmorillonite mixture. The main contribution of the article according to the authors is that it's the first time that two different clays in combination with cellulose have been rigorously studied for hydrogel formation. However, there are the following comment.

1. The author claimed that "this work presents a mechanism for modifying rheological properties using renewable and environmentally-friendly nanocellulose and clays which could be used in home and personal care formulations". However, the article did not carry out relevant experiments to demonstrate its mechanism, reproducibility, environmental friendliness and biocompatibility. It was suggested to characterize the composition, structure and other properties of composite.

We thank the reviewer for their comments and feedback. The above-mentioned claim is based on the data provided in the article (showing that rheological properties can be modified), the replicate

samples (showing reproducibility) and the existing data on the environmentally friendly nature of the components. The biocompatibility of the clay components has been further emphasising by adding the following sentences to the introduction with appropriate references:

(page 4 lines 76-77) “These clays have low toxicity, even with oral ingestion, and so are ideal candidates for biocompatible product development.[23, 24]”

(page 4 lines 68-69) “Furthermore, OCNF has been shown to be biodegradable.[22]”

2. The rheological properties of shear-thinning gels are affected by three factors: OCNF concentration, clay concentration and clay type. How did authors to design mentioned above three parameters to affect the rheological properties of gels.

The effect of the three above-mentioned properties on the rheological behaviour of gels were tested in the following ways:

OCNF Concentration – The OCNF concentration was varied while keeping the concentration of clays constant. The change in the rheological properties of the gels with changing OCNF concentration demonstrates how OCNF influences this behaviour.

Clay Concentration – The concentration of each clay was varied while keeping the concentration of OCNF constant. The change in the rheological properties of the gels with changing clay concentration demonstrates how the clays influences this behaviour.

Clay type – By comparing the rheological behaviour of the gels at equitable fractions of each clay type (with the OCNF concentration kept constant) we are able to demonstrate the differential effects that these two clays have on the rheological properties.

To clarify this, the following sentence has been added to the end of the methods section:

“Experiments were designed to test the effects of OCNF concentration, clay concentration and clay type by varying each factor in turn while the others were kept constant.”

3. In “Figure 1”, the author claimed that “it is clear that the montmorillonite suspension has a stronger frequency dependency than the Laponite suspension.” It needs further explanation and experimental evidence, or documentation.

The authors thank the reviewer for their feedback. It is common in rheological analysis to examine frequency sweeps for ‘frequency dependence’. This is where the storage and loss moduli change with changing frequency (as is shown to occur for montmorillonite but not Laponite in Figure 1). The above-mentioned sentence has been modified to clarify this data for those that may not be familiar with rheological analysis: (page 7 line 153-155)

“From the graph it is clear that the montmorillonite suspension has a stronger frequency dependency than the Laponite suspension, as demonstrated by the increase in both the storage and loss moduli with increasing angular frequency”

4. In “Figure 2”, the author claimed that “Laponite contributed more significantly to the solid-like behaviour and that further addition of OCNF had little effect.” and “Laponite is a much smaller particle and therefore on a per-weight basis has a higher contribution to electrostatic interactions than OCNF fibrils”. It is difficult to get this conclusion directly from “Tan(δ) values of mixtures of Laponite and montmorillonite with OCNF”. The author's statement needs further experimental proof.

The paragraph beginning on line 172 details the physical meaning of tan(δ) (i.e. an indication of solid vs. liquid behaviour). Therefore, the data presented in Figure 2 – which shows the tan(δ) values for different combinations of OCNF and clay is itself evidence for the different influences of Laponite, montmorillonite and OCNF on rheological behaviour.

The tan(δ) changes more upon addition of Laponite than upon addition of montmorillonite. This is experimental proof that it contributes more significantly to the solid-like behaviour. The same is true for the other analysis made regarding Figure 2.

5. In “Figure 2”, the physical quantity on the abscissa lacks a logical relationship. It is recommended to redraw the diagram.

We disagree that the quantities on the x-axis lack a logical relationship. The X-axis shows the OCNF content and clay content allowing easy comparison between the different suspensions investigated. They are logically linked by the experimental design referenced above.

6. Please give more description of result, the equation or reference to calculate some index etc. to help readers to understand.

The authors apologise but we do not understand what the reviewer is asking for here. The comment is very vague and we do not understand which index or calculation are they looking for, or which particular section of the results they are referring to.

7. The abbreviation should be given its full name when it first appears.

The manuscript has been carefully reviewed to ensure that all abbreviations are properly defined at their first usage.

Reviewer #2: The manuscript described the rheological modification by two different clays (Laponite and montmorillonite) in combination with cellulose nanofibers, and further the effect on hydrogel formation. The manuscript did a systematically study, and a detailed discussion of the rheological data. Before its consideration for publication, minor revisions are needed according to the following comments.

1. Please give a detailed description for the preparation of OCNF.

A description of the preparation method has been added to the supporting information.

2. "the OCNF was freeze dried and then resuspended in deionised water to 2 wt%", did the OCNF can be redispersed well without any sediments? As we know, the redispersing of dried cellulose nanofiber is still a big difficult.

The reviewer is right in that dispersing cellulose nanofibers can be difficult. However with sonication as outlined in the methods section we were able to redisperse the fibrils without sedimentation. A comment to this effect has been added to the experimental section on page 5 line 106:

"This allowed redispersion of the fibrils without sedimentation."

3. Page 6, line 165-168, the author mentioned that "aging effect" could be observed for the combinations of Laponite and montmorillonite with OCNF in previous reported literature. If so, how about the combination of montmorillonite with OCNF? Are there any literatures, or any speculations from authors?

The authors thank the reviewer for this insightful comment. To properly answer it we would like to separate it into two parts. Firstly, the aging effect occurs for both Laponite and montmorillonite mixed with OCNF, as demonstrated by Figure S22. The sentence mentioned by the reviewer is a possible explanation for why our rheology results aren't quite the same as that reported by the previous paper – that is, measurements were performed at different time points. To clarify this point we have expanded the paragraph to include the following sentence:

"We show the evolution of rheological behaviour in S22 Fig and this evolution is expected to continue with increasing time."

In regards to the second part of the query, for montmorillonite mixed with OCNF, to the authors' knowledge, that mixture has not been rigorously studied in the literature.

4. It is better to provide SEMs to observe the OCNF networks and the combination state with clays

The authors do not agree with the reviewer that SEM observations would be good for further studying the arrangement of OCNF and clay particles. SEM would necessitate a dried system and in our experience the physical arrangement in the dried state is quite different to that in the wet/gelled state, even when using supercritical drying methods. The solids content of these gels is low, and the structures found in solution are not maintained when the gels are dried. We also did not do cryo-TEM because we expect the clay components to prevent transmission of electrons and obscure the cellulose fibrils.

Reviewer 3

The authors present an interesting analysis of a mixed organic/inorganic gel comprising of oxidised cellulose nanofibres with two different types of clay particle. The system is probed with both rheometry and small-angle X-ray scattering, looking at both the structures that the fibre-clay gel forms and the flow properties of the gel/yielded fluid. The manuscript tells an interesting story that is suitable for PLOS One and will be more satisfying to the reader with some additional polishing as described below.

Comments

1. The origin of the interaction forces between the clay particles and the OCNFs needs to be described in the introduction so that the experimental data makes sense. It is established that all particles are negatively charged with the clays perhaps having a positive edge charge in some circumstances. The reader is left to assume that these are attractive gels (and not a repulsive gels) until some distance into the results before is it postulated that the positive edges of the clays is just attractive enough. Perhaps the authors could describe the gelation mechanisms a little better in the introduction.

The authors thank the reviewer for this thoughtful feedback. We have added the following paragraph to the introduction to better preface the mechanisms of gelation (page 4 lines 79-87) :

"The charged nature of the clay particles provides two potential avenues for interaction with the OCNF fibrils. Specifically, the positively charged edges of the particles will have attractive interactions with not just the negatively charged faces of other particles, but also the negatively charged OCNF fibrils, thus building an inter-connected network. Furthermore, the negatively charged faces of the particles may contribute to exclusion/repulsive interactions with the OCNF fibrils which will also contribute to the rheological properties of the gels. A previous study examining mixtures of Laponite and OCNF highlighted the importance of clay concentration to the relative importance of clay-clay vs. clay-fibril interactions for rheological properties.[18]"

2. Is there a role for shape in giving an attractive depletion potential, given the size and aspect ratio differences; this is a potentially (pun intended) extremely interesting aspect (pun intended) of this work given that the fibres have extremely large aspect ratios and are also so much better than the clay particles. Depletion potentials based on size/shape differences were demonstrated by Cinacchi et al. with both simulations and experiments.

The authors thank the reviewer for bringing this interesting facet of the work to our attention. Unfortunately exploring the specific effect of aspect ratio and depletion forces is beyond the scope of this study. However, we have added the following to the conclusion (including reference to Cinacchi) to highlight the area for future study (page 21 line 468-470):

“The effect of particle shape on these interactions, for example the influence of aspect ratio on depletion forces,[36] would be a valuable area for future study to better understand the interactions of differently shaped particles.”

3. The authors mention that the mesh size of the OCNFs might vary with salt but do not probe any salt effects. If an electrostatic interaction is needed for the gelation then salt is quite important. Can the authors point to suitable literature for this?

The authors thank the reviewer for this comment.

Salt has been shown to affect gelation of OCNF, as highlighted by previous work from our group in which we made an extensive study of the effects of salt on the OCNF network (doi: 10.1039/C8CP00355F.). As with salt, the clay particles will have electrostatic interactions with the OCNF.

References to the previous work on salt with OCNF are provided on page 8, 9, 17 and 19 (reference 3).

4. p13 L354 “As shown in Figures S24 and S25 ...”. The assertion that these figures justify a simple addition of the individual scattering components is lost on this reviewer; fits do not appear to be shown on those two figures at all. Indeed, fits do not appear to be included for a any of the SAXS data past Fig S23.

The authors apologise for this oversight. The sentence should have read “As shown in Figure 6”. The text has now been corrected.

The reviewer is correct in that fits have not been provided for SAXS data past Fig S23. This is because the fits are largely unchanged for different clay/OCNF concentrations and do not reveal further information about interactions or mechanisms of gelation. The remaining SAXS data is provided only for reader interest.

5. The SAXS data is comparatively featureless and yet the model used would have around 10 free parameters. The SAXS analysis could be more strongly presented if more details of the models were given in the SI, including which parameters were permitted to vary and which were held fixed based on extant data or previous experiments.

The authors appreciate the reviewer’s suggestion, and the following has been added to the S1 Supplementary information:

“Fitting was performed by fixing the SLDs of both the solvent and the particles at calculated values. The length of the OCNF cylinders was also fixed to 1000 Å as their true length is outside of the measurement range of this SAXS instrument. The background value for all fitting was fixed at the

intensity value of the plateau at high q. For all fitting the scale factor was allowed to vary between 0.01 and 0.5 but in all cases ended being ~0.02-0.07.

For fitting the OCNF systems, the minimum radius and axis ratio were allowed to vary. For fitting the clay systems, both the length and the radius were allowed to vary. However, it was found that radii always fell above 250 Å for Laponite and 1000 Å for montmorillonite and did not affect the length. Therefore the maximum number of parameters fitted for any system was 3."

6. The volume fraction of the OCNFs must be quite large to have a 30nm pore size in the gel; what volume fraction does this correspond to? How does this volume fraction correspond to the dilute approximation that is used in the SAXS analysis where there is no structure factor included? While adding an additional 2-7 free parameters for a structure factor would not enhance the believability of the analysis, it seems unlikely that one is not needed.

The authors agree with the reviewer that additional parameters would not help with the SAXS analysis. The SAXS data did not indicate the need for a structure factor to fit the data and so one has not been included, as this would include extra free parameters in the fit which are not justified. The length scale of this structural interaction may be outside of the probed range of the SAXS.

The density of cellulose is not far from one, therefore the volume fraction is similar to the weight fraction (0.75 to 2wt%). The mesh size measured in the cited paper is based on cryo-TEM images of gels which necessitates calculating a 3D structure from a 2D image. For these reasons, the actual mesh size may be larger than that reported in the cited paper.

7. The purpose of figures S24-S32 is unclear – these look to be interesting data but nothing seems to be done with them. There is minor reference to them at L361 but this probably needs to be expanded to an additional paragraph with systematic analysis of the data – if the scattering is genuinely additive as claimed then it should be relatively easy to extract some clustering or ordering parameters from these data.

The authors thank the reviewer for this suggestion however, we are uncertain which clustering or ordering parameters the reviewer is referring to. In all mixtures studied here, the clays are very dilute and therefore the nematic ordering that might occur in concentrated clay suspensions will not occur. The SAXS data presented here can be described via purely additive scattering from the two components, with no other parameters required and therefore the only parameters that can be extracted are related to the physical properties of the components, which are shown in Table S2.

8. This reviewer would love to see some additional effort made to link the structures and rheology that is contained in this manuscript. A little more explanation to help the reader understand how the specific structures measured by SAXS are driving the observed rheology would help the reader better understand the impact of this work. There's a sentence that is

doing this in the conclusions; and additional paragraph and perhaps a schematic for the end of the discussion would improve the manuscript.

The authors would like to thank the reviewer for their suggestions. The SAXS results highlight that at least in these dilute systems, interactions are on length scales that are not accessible by SAXS. To better highlight this, the following sentence has been added to page 18 of the discussion:

"These results further confirm that in these dilute systems, the interactions between OCNF and clay particles are happening at length scales that are not accessible by SAXS."

In addition, Figure 7 has been added which illustrates the interactions of Laponite and montmorillonite with OCNF.

Given this is journal does minimal typesetting, the authors are encouraged to:

9. p6 L137 & L139. Rewording this sentence so that it doesn't start with "tan(δ)" is needed. (also in a few other places)

The manuscript has been thoroughly checked and all sentences rewritten so that they don't start with tan(δ).

10. Throughout, ensure that the prime and double prime in G' and G'' are not curly quotation marks.

All instances of curly quotes for prime have been replaced with straight quotes.
