# Supplementary material for: Rheological modification of partially oxidised cellulose nanofibril gels with inorganic clays
Source: PLoS One. 2021 Jul 7;16(7):e0252660. doi: 10.1371/journal.pone.0252660 (PMC8263268; doi:10.1371/journal.pone.0252660)
Supplement: Attachment — Submitted filename: Response.pdf [file pone.0252660.s003.pdf]

Prof. Karen J. Edler
Department of Chemistry,
Bath BA2 7AY • United Kingdom

Telephone 01225 384192
Email: K.Edler@bath.ac.uk

Dear Pratheep K. Annamalai,

Thank you for giving us the opportunity to resubmit our manuscript titled "Rheological modification of partially oxidised cellulose nanofibril gels with inorganic clays" with further feedback from the reviewers.

We were glad to see that Reviewer #3 felt that we had adequately addressed the comments. The further concerns of Reviewer #1 have been addressed below (our responses are shown in red). We have also made adjustments following your editorial comments.

We hope that the paper is now ready for publication in PLOS ONE and are confident that it will be of interest to your readership.

Kind Regards,

Karen J Edler

Karen J Edler

Professor of Soft Matter

Additional Editor Comments (if provided):

Authors are appreciated for revising the manuscript carefully. Further minor revision is required for improving clarity and flow of presentation.
1. Fig. X is okay for single figure. For more than one figures, it is better to cite as 'Figures' or Fig. 1 and 2, instead of 'Figs'.

All cases of 'Figs' have been replaced with Figures throughout the manuscript. However, we would like to mention that this is in opposition to the written formatting requirements that you linked us to during the previous round of review; a screenshot of that advice is given here:

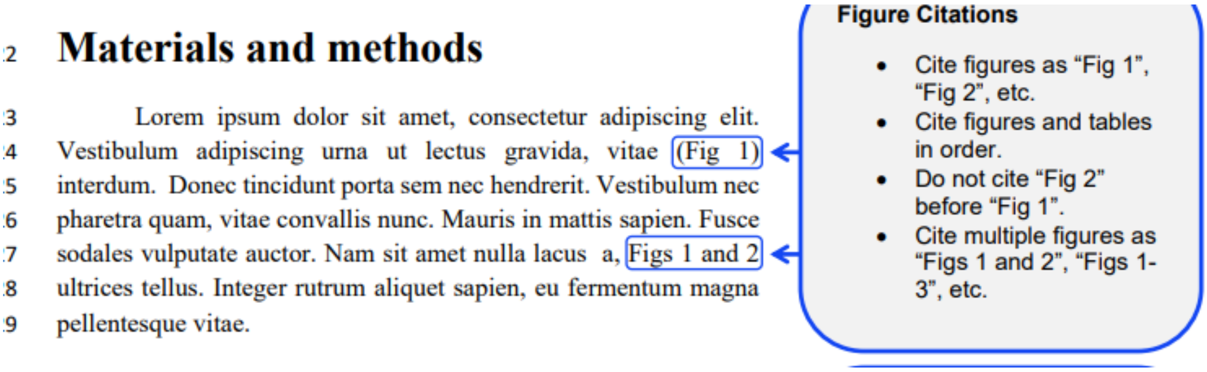
Materials and methods

Lorem ipsum dolor sit amet, consectetur adipiscing elit. Vestibulum adipiscing urna ut lectus gravida, vitae (Fig 1) interdum. Donec tincidunt porta sem nec hendrerit. Vestibulum nec pharetra quam, vitae convallis nunc. Mauris in mattis sapien. Fusce sodales vulputate auctor. Nam sit amet nulla lacus a, Figs 1 and 2 ultrices tellus. Integer rutrum aliquet sapien, eu fermentum magna pellentesque vitae.

Figure Citations

- Cite figures as “Fig 1”, “Fig 2”, etc.
- Cite figures and tables in order.
- Do not cite “Fig 2” before “Fig 1”.
- Cite multiple figures as “Figs 1 and 2”, “Figs 1-3”, etc.

2. All the supporting information can be in one file (if possible). The citation of supporting information parts can be consistent such as "Figure SX or Table SX or Page SX in Supporting Information. Please insert page numbers for Supporting information file. Accordingly the figure captions can be revised.

The supporting information has been condensed into one file.

Page numbers have been added to the SI file.

Reviewer #1: 1. For comment 1, it still lack some experimental evidence or reference to confirm the mechanism for modifying rheological properties using clay, e.g. SEM or TEM to reveal the distribution of clay particles in OCNF gel, FTIR or Laser Raman or XPS to demonstrate the interactions between OCNF and clay platelets.

The authors appreciate the reviewer’s concern. However, most of the above-mentioned techniques are inappropriate for this system and will not provide the information that the reviewer seeks. For example, the OCNF particles are too small to view in SEM and can only be visualised in TEM. However, the electron density disparity between clay particles and OCNF means that the distribution of clay within OCNF will be impossible to see using TEM as the OCNF will be essentially invisible. TEM images of OCNF are acquired by staining using a high electron density salt – however addition of salt will irreversibly alter any interactions present between clay particles and OCNF, thus destroying the information that is sought. OCNF samples for TEM are also typically diluted even further than in our solutions to avoid aggregation, allowing individual fibrils to be imaged on the grid – this would alter the interactions between any species present so they would not be representative of the distributions giving rise to the rheological behaviour reported. In addition, both SEM and TEM will require high vacuum, so the samples will need to be completely dried out. Even using supercritical drying, in such high-water-content systems, the structures are irreversibly altered by removal of water, meaning that the desired information on the distribution of clay particles within the OCNF network in the wet gels cannot be found. Thus, these two techniques are unsuitable to provide information on the distribution of clay particles in the OCNF gel network.

FTIR or laser raman techniques are useful techniques when a chemical reaction has occurred, altering the types of bonding present in the system so that patterns before and after the reaction can be compared. However here no chemical reactions occur between our components, and thus we do not expect large changes in the spectra using these

techniques. In addition, the suspensions of clay and cellulose fibrils are rather dilute at maximum concentrations of 2wt% each, so the water signals dominate in the measurements of these systems, obscuring any tiny changes due to physical interactions between the components. FTIR spectra for the two separate components and their mixtures at equivalent concentrations in water are shown as an example below in Figure 1, for the highest concentrations studied, however this adds no useful information to characterise the interactions between the clay and OCNF in the wet gels – the spectra of the mixtures are clearly merely the sum of those from the two components. Thus so we do not feel that to adding them to the manuscript provides any useful information.

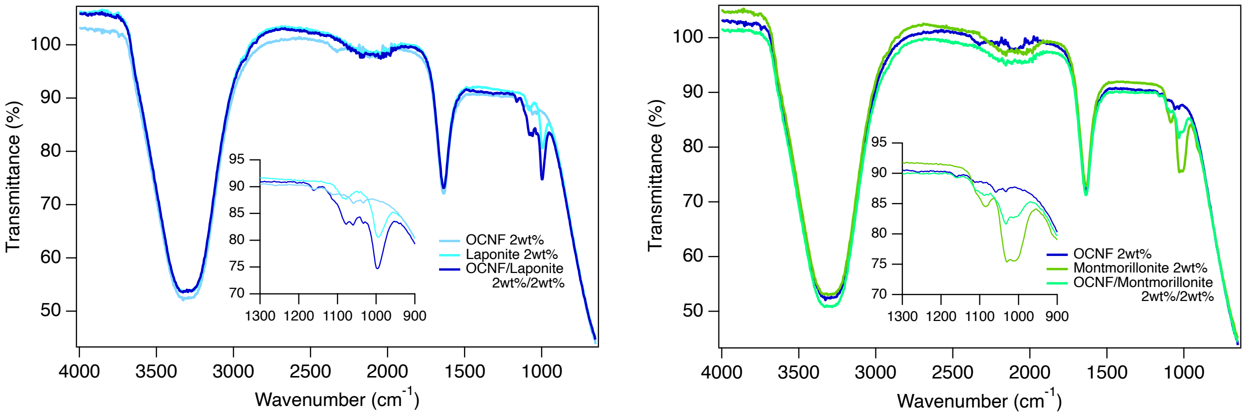

Figure 1: FTIR data from (left) OCNF/Laponite gels and (right) OCNF /Montmorillonite gels. Inset in both figures is an expanded view of the region between 900-1300cm^{-1} showing the additive nature of the spectra.

We are also puzzled by the request for XPS measurements since this technique provides information about the elements present in a sample, specifically at the sample surface, so no bulk information on interactions can be obtained using this method – yet the interactions which modify the rheology in this system are occurring in the bulk. We also are not carrying out any chemical reactions in this system, simply physical mixing and so the particle compositions remain unchanged from those of the component materials, which are already well characterised by us and other researchers, as noted in the text. Additionally, measuring XPS on aqueous liquids requires highly specialised instrumentation since this technique also normally requires UHV conditions. Thus we are unable to carry out XPS measurements on our wet gel systems since we do not have access to such specialised equipment, however given it will only probe the liquid surface, it will also not provide the required information on interactions between clay and OCNF particles in the bulk.

The purpose of this paper was to demonstrate the effect of combining clay particles and OCNF on the viscosity of the formulation, and the rheological data is more than adequate for that. SAXS highlights that there are no direct interactions which altering the structures of the particles at the length scales measured, and thus that physical interactions on relatively large length scales are responsible for the changes in viscosity. Further characterisation of the underlying mechanisms is difficult due to the nature of the systems studied, and so is beyond the scope of the current paper.

2. For comment 6, it’s means that it hard to understand Line 278 to 288. What is “The exponent (flow behaviour index)”,” The base number (flow consistency index)”. It is suggested to give more description of result, the equation or reference to calculate some index etc. to help readers to understand.

The authors thank the reviewer for clarifying their question.

The authors would like to direct the reviewer to the supporting information (page 15-16) where the power law model was already described in more detail.

However, to further clarify this methodology, Figure S22 has been added to the supplementary information (page16) which shows power law fits to some of the rheological data. We have also modified the text in the main document to include the general form of the power law equation, which was used to calculate the values discussed and added an appropriate citation to the literature. We have also added some of the values obtained from the fitting to the text to further clarify the discussion. The text (lines 262-275) now reads:

“Fitting models to the linear portion of flow curves, using the power law $\eta = K\dot{\gamma}^{m}$ (where h is viscosity, $\dot{\gamma}$ is the shear rate, K is the flow consistency index and m is the flow behaviour index) can give quantitative information about the systems for comparison [32] (see also description in SI, page 15-17). Table S1 shows the best-fits for each combination of Laponite or montmorillonite with OCNF. The exponent (flow behaviour index) was similar for most mixtures of OCNF and Laponite, with values around 0.1, although it decreased with increasing Laponite concentrations, suggesting more prominent shear thinning behaviour. Mixtures with montmorillonite were much the same, also having values for the exponent around 0.1, except with 0.75 wt% OCNF, where the exponents were much closer to 1, suggesting much less shear thinning occurs in these low viscosity mixtures than in the other systems. The base number (flow consistency index) which is a measurement of the ‘thickness’ of a system followed the same patterns as those of viscosity and tan(δ) discussed above (see Table S1), increasing with increasing clay or OCNF concentrations and being much higher for systems with Laponite than montmorillonite, as expected from the other measurements.”

Reviewer #3: The authors have done an excellent job in improving this manuscript based on the comments and suggestions provided by each of the reviewers. They have certainly adequately address all of this reviewer's comments and even appeared to take the bad puns in their stride. This is a delightful piece of work and I look forward to see it in in PLOS ONE.

The authors would like to thank the reviewer for their thoughtful comments (puns included) as well as their time.
